# Assessing Splicing Variants in the *PAX6* Gene: A Comprehensive Minigene Approach

**DOI:** 10.1111/jcmm.70459

**Published:** 2025-03-25

**Authors:** Kseniya Davydenko, Alexandra Filatova, Mikhail Skoblov

**Affiliations:** ^1^ Department of Functional Genomics Research Centre for Medical Genetics Moscow Russia

**Keywords:** Aniridia, minigene, *PAX6*, retinal diseases, splicing

## Abstract

Haploinsufficiency of the *PAX6* gene causes aniridia, a congenital eye disorder characterised by the absence or malformation of the iris and foveal hypoplasia. Previous studies indicate that pathogenic splice variants account for up to 15% of all disease‐causing *PAX6* variants. However, this proportion may be significantly underestimated because the pathogenicity of splice variants can only be accurately established through experimental validation. In this study, we developed and validated a system of eight minigene constructions for the functional analysis of splicing variants in the *PAX6* gene. This system covers all *PAX6* coding exons and allows the analysis of any exon and most intronic variants of *PAX6*. Our comprehensive approach, employing fragment analysis and deep targeted sequencing, enabled us to accurately characterise 38 previously described *PAX6* variants, including challenging cases with multiple splicing events. The application of our system revealed that the number of pathogenic splicing variants might be closer to 30% of all pathogenic *PAX6* variants. This finding considerably reshapes our understanding of their significance in the genetic landscape of aniridia.

## 1 Introduction

The *PAX6* gene, located on chromosome 11p13, encodes a highly conserved transcription factor that acts as a master controller of eye development [1]. The 422 amino acid PAX6 protein contains a DNA‐binding paired domain (PD) and paired homeodomain (HD) separated by a flexible linker, along with a C‐terminal region enriched with proline–serine–threonine (PST), which acts as a transcriptional activation domain [2, 3, 4, 5, 6]. *PAX6* is composed of 14 exons, and its expression is controlled by three independent promoters, producing a number of alternative transcripts [7, 8, 9]. The vertebrate *PAX6* gene also expresses a third isoform, PAX6ΔPD, which lacks PD and is produced by the internal promoter Pα [8, 10, 11]. Despite its unknown physiological roles, PAX6ΔPD functions differently from PD‐containing *PAX6* isoforms, and its overexpression disrupts lens and corneal development [12].

Pathogenic variants in *PAX6* lead to a spectrum of eye abnormalities. The most prevalent phenotype is congenital aniridia, characterised by the absence or underdevelopment of the iris, nystagmus, foveal hypoplasia and other ocular complications leading to reduced visual acuity [13, 14]. Aniridia is typically inherited in an autosomal dominant fashion, but atypical cases associated with other genes and different inheritance patterns have also been reported [15, 16]. Despite the high penetrance of *PAX6* haploinsufficiency, phenotypic variability is common even among carriers of the same variant [17]. Other *PAX6*‐associated disorders include optic nerve anomalies, anterior segment dysgenesis (ASD), microphthalmia and related conditions such as corneal issues, cataracts and glaucoma [18, 19]. Systemic manifestations include neurodevelopmental disorders, such as autism and mental retardation, as well as pineal and pituitary gland abnormalities, obesity and diabetes mellitus [20, 21, 22, 23, 24, 25, 26].

More than 700 pathogenic variants in the *PAX6* gene have been identified thus far [27]. A significant portion of these variants results in premature stop codons through nonsense, frameshift, or splicing errors, leading to haploinsufficiency of the PAX6 protein [28, 29]. Missense variants are predominantly located in the paired and, less commonly, homeobox domain, highlighting their critical role in *PAX6* function [30, 31, 32]. Splicing variants account for up to 15% of all pathogenic variants in the *PAX6* gene [33, 34]. Most of them are identified at terminal AG‐GT dinucleotides of canonical acceptor (3′SS) and donor (5′SS) splicing sites [34]. Splice‐affecting variants were also found in noncanonical splice site nucleotides, regulatory elements, deep intronic sequences and the 5′ and 3′ untranslated regions (UTR) of the *PAX6* gene [34, 35, 36, 37, 38].

In recent years, the use of massively parallel sequencing methods has significantly improved the diagnosis of inherited diseases. However, despite the significant increase in the number of variants identified, their accurate clinical interpretation is often challenging. While there have been advances in silico prediction tools, functional studies remain critical to accurately assess the impact of identified variants on splicing [39]. Direct analysis of RNA represents the optimal methodology for the assessment of splicing variants. However, the acquisition of samples from patients may prove challenging due to the tissue‐specific expression of *PAX6*. Minigene splicing assays offer an effective alternative [35, 40]. In recent years, a number of studies have employed minigene‐based analyses to investigate the splicing variants of *PAX6*. However, there have been no large‐scale studies of the aberrant splicing of *PAX6* to date.

In this study, we evaluated the impact of intronic and exonic SNVs on the splicing of all described pathogenic *PAX6* variants. By employing a system of eight minigene constructions covering the entire coding sequence, we assessed the effects of exonic SNVs, variants in canonical and noncanonical donor and acceptor splicing sites and deep intronic variants located in our expression vectors. This minigene system successfully elucidated the molecular pathogenicity of previously described SNVs in the *PAX6* gene identified in patients with aniridia.

## 2 Materials and Methods

### 2.1 Variant Nomenclature and Accession

Single nucleotide variants (SNVs) were named according to the Human Genome Variation Society (HGVS) recommendations [41]. The hg19 assembly and NG_008679 were used as the reference genome sequences. NM_000280.4 was used as a reference transcript.

### 2.2 Bioinformatic Analysis

The effects of SNVs on pre‐mRNA splicing were predicted using SpliceAI (Illumina, USA) [42] and MaxEntScan (MIT, USA) [43] tools. The variants were annotated using the wANNOVAR tool (Wang Genomics Lab, USA) [44]. The SVM‐BPfinder tool (Universitat Pompeu Fabra, Spain) [45] was used to predict the branch point.

### 2.3 Minigene Constructions

We used the pSpl3‐Flu2‐TK vector to create minigene plasmids. This vector was derived from the previously described pSpl3‐Flu vector [40] by deleting the cryptic splicing sites within the HIV‐tat intron and replacing the strong CMV promoter with the weaker HSV‐TK promoter. The use of the TK promoter led to the expression of minigenes at a level closer to the physiological level.

Wild‐type minigene plasmids were prepared using target exons and variable flanking intronic sequences of the *PAX6* gene amplified from control genomic DNA using Q5 High‐Fidelity DNA Polymerase (NEB, USA). The PCR products were cloned and inserted into the pSpl3‐Flu2‐TK‐del vector by the restriction digestion method using XhoI/SacI and BamHI/XbaI sites.

Mutant minigene plasmids were obtained by the introduction of selected SNVs into wild‐type plasmids via single‐primer site‐directed mutagenesis [46]. The nucleotide sequences of the plasmids were confirmed using Sanger sequencing.

All primers used for cloning and mutagenesis are listed in Table S1.

### 2.4 Minigene Splicing Assay

Human embryonic kidney 293 T (HEK293T) cells were cultured in high‐glucose DMEM supplemented with alanylglutamine (PanEco, Russia) and 10% foetal bovine serum (PanEco, Russia) in 5% CO_2_ at 37°C. Twenty‐four hours before transfection, 24‐well plates were treated with poly‐L‐lysine and 6 × 10^4^ cells were seeded in each well. Two hours prior to transfection, the growth medium was replaced with DMEM without FBS. Samples containing 750 ng of wild‐type or mutant plasmids were transfected into HEK293T cells using the calcium phosphate transfection method [47]. Transfection efficiency was assessed using a flow cytometer and the FloMax software package (Partec, Germany).

Forty‐eight hours after transfection, the cells were harvested and total RNA was isolated using Extract RNA reagent (Evrogen, Russia) according to the manufacturer's recommendations. The isolated RNA was treated with DNase I (Thermo Fisher Scientific). cDNA was synthesised using the 5X RT MasMIX of recombinant MMulV H Reverse Transcriptase (Dialat, Russia). The mRNA structure was determined by PCR using SmarTaq DNA polymerase (Dialat, Russia) and plasmid‐specific primers (Table S1). The thermal cycling conditions were as follows: initial denaturation at 95°C for 5 min; 35 cycles of 95°C for 15 s, annealing at 62°C for 15 s and extension at 72°C for 15–30 s; followed by a final elongation at 72°C for 5 min. The experiments were carried out in two biological replicates. The PCR products were analysed using denaturing urea polyacrylamide gel electrophoresis, followed by Sanger sequencing.

### 2.5 Fragment Analysis

To perform fragment analysis, cDNA was amplified using a 6‐FAM‐labelled plasmid‐specific forward primer (Table S1). PCR was performed using HF‐Fuzz DNA polymerase (Dialat, Russia) and consisted of an initial denaturation at 98°C for 5 min, followed by 27–30 cycles of 98°C for 10 s, 62°C for 10 s (annealing) and 72°C for 15 s (extension), with a final extension step at 72°C for 5 min. The products were separated on an Applied Biosystems 3500/3500xL Genetic Analyser capillary electrophoresis system (Thermo Fisher Scientific, USA). The relative quantities of the isoforms were assessed from the peak heights of each isoform compared to the total area under the curve and the peak height for all isoforms for each sample. Measurements were performed in at least two repetitions.

### 2.6 Targeted Next‐Generation Sequencing (NGS) of PCR Products

The PCR products for NGS were obtained by PCR using SmarTaq DNA polymerase (Dialat, Russia) and plasmid‐specific primers as described above for fragment analysis. NGS libraries were prepared using the ‘SG GM’ Kit (Raissol) and sequenced on the FASTASeq platform in a paired‐end mode (2 × 150 bp). The targeted loci have a range of coverage from 100 (in one case) to over 3,000,000x. The raw sequencing data were processed with a custom pipeline based on open‐source bioinformatics tools. In brief, the pipeline involved quality control of raw reads using the FastQC tool v0.12.1, followed by read mapping to the minigene plasmid sequences and sorting using STAR 2.7.11a. Splice junctions were visualised using Sashimi plot in the IGV browser.

## 3 Results

### 3.1 Analysis of Potentially Splice‐Altering SNVs in the *PAX6* Gene

**FIGURE 1:**
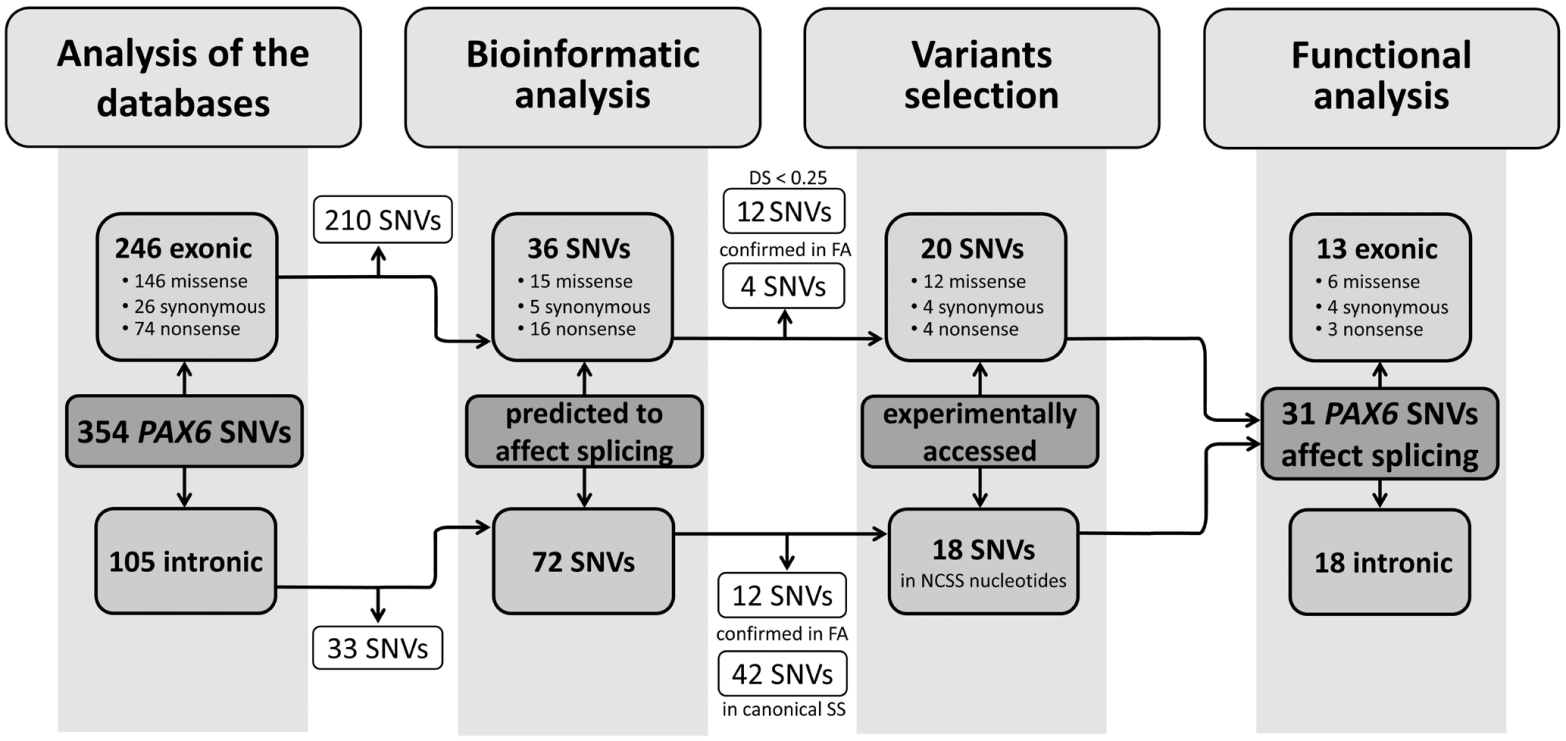
Flowchart for studying splicing variants in the *PAX6* gene. In this study, all unique single nucleotide variants in the *PAX6* gene reported in the HGMD, ClinVar and LOVD databases were in silico analysed for their potential impact on splicing. A total of 18 intronic and 20 exonic variants were selected and tested via a minigene expression system containing all coding exons of the *PAX6* gene. Abbreviations: SNV, single nucleotide variant; DS, SpliceAI Delta score; FA, Functional analysis; SS, Splicing site; NCSS, noncanonical splice site.

It is known that the contribution of splicing SNVs to the spectrum of pathogenic variants associated with various diseases is often underestimated. In addition to noncanonical splice site nucleotides, such variants can be located in regulatory sequences, deep in introns and also in exons, masquerading as synonymous, missense, or nonsense variants. Therefore, we first attempted to assess the total number of potentially splicing‐altered *PAX6* variants. For this purpose, all *PAX6* single nucleotide variants (SNV) registered in the HGMD Pro 2022.1, LOVD v.3.0 and ClinVar databases (from 27‐05‐2022) were screened. In total, 354 unique SNVs were found, with 249 SNVs located in exons and 105 in introns of the *PAX6* gene (Figure 1).

The SpliceAI algorithm was used to assess the probability of the influence of the selected variants on splicing. SpliceAI evaluates the pathogenicity of variants using the parameter Delta Score (DS), which ranges from 0 to 1 and can be interpreted as the ability of the variant to influence splicing. Variants with a DS value > 0.2 are considered likely pathogenic [42]. SpliceAI analysis revealed that 36 exonic and 72 intronic SNVs in the *PAX6* gene have splice‐altering potential (Figure 1).

Analysis of the 72 intronic variants registered in the databases and predicted by SpliceAI as potentially splice‐altering revealed that only 12 of them were previously confirmed by functional studies. Among the remaining 60 variants, 18 intronic variants are located outside the canonical splicing dinucleotides (±1, 2) and were registered in the databases but had not been confirmed by functional studies, so we selected them for further analysis (Table 1). Of these variants, nine SNVs were located in the acceptor splicing site (Positions −3, −5 and − 9), and seven variants were located in the donor splicing site (Positions +3, +4 and + 5). Two variants (c.357+136G>A and c.357+334G>A) were located deep in intron 6 of the *PAX6* gene. According to SpliceAI prediction, 15/18 variants had a delta score > 0.2 (high recall/likely pathogenic). Among them, three variants had a delta score > 0.5 (recommended/pathogenic) and seven variants had a delta score > 0.8 (high precision/pathogenic). The delta scores of the three variants (c.142‐3C>G, c.917‐3C>G and c.1183+4A>G) were slightly lower, but still close to 0.2 (0.19, 0.16 and 0.17, respectively).

**TABLE 1:** Potentially splice‐altering *PAX6* variants selected for minigene splicing assay.

Variant.	Reference	Disease	Details	ACMG‐AMP
**Intronic SNVs**				
c.141+3G>C	ClinVar: 460458	Aniridia	Allele origin: germline	Uncertain significance (PM2, PP3)
c.141+4A>T	ClinVar: 800401; PMID: 8111379, 2,360,764	Aniridia	Familial case is reported.	Likely pathogenic (PS4, PM2, PP1, PP3, PP4)
c.142‐5T>G	ClinVar: 430981; PMID: 28321846, 30,315,214	Aniridia	PMID 30315214 describes a proband with iris hypoplasia without nystagmus, macular hypoplasia, congenital cataract, strabismus and emotional lability. His mother has partial aniridia without nystagmus, macular hypoplasia, cataract, keratopathy, ptosis, epicanthus and vitreous body destruction.	Likely pathogenic (PM2, PS2, PP1, PP4, PP3)
c.142‐3C>G	PMID: 16785853, 25,525,159	Aniridia	Familial case is reported.	Uncertain significance (PM2, PP3, PP1)
c.357+4A>T	ClinVar: 460459	Aniridia	Reported de novo status.	Likely pathogenic (PM2, PP3, P5, PS2)
c.357+5G>A	ClinVar: 800429; PMID: 18483559, 32,360,764, 34,101,622	Aniridia	PMID 34101622 reported two familial cases of complete aniridia with foveal hypoplasia, nystagmus, glaucoma and cataract. Proband I also has asthma, and MRI showed a small posterior corpus callosum and anterior commissure. Proband II has obesity, hypercholesterolemia and irritable bowel syndrome.	Likely pathogenic (PS4, PM2, PP2, PP3)
c.357+5G>C	PMID: 32467297	Aniridia	Sporadic case is reported.	Likely pathogenic (PM2, PM6, PP3, PP4)
c.357+136G>A	ClinVar: 559620; PMID: 30291432	Isolated aniridia	Reported de novo status.	Likely pathogenic (PM2, PS2)
c.357+334G>A	ClinVar: 559621; LOVD: PAX6_000889; PMID: 30291432	Isolated aniridia	Inheritance is unknown.	Uncertain significance (PM2)
c.683‐9C>G	ClinVar: 800449; PMID: 22361317, 32,360,764	Aniridia	PMID: 22361317 reported a mild aniridia phenotype with minimal nystagmus, mild limbal keratopathy, mild foveal hypoplasia and no lens opacities. Familial cases are also reported.	Likely pathogenic (PS4, PM2, PP1, PP2, PP3, PP4)
c.683‐5T>C	ClinVar: 800450; LOVD: PAX6_000810; PMID: 32360764, 34,942,114	Aniridia	PMID 34942114 reported a familial case of mild atypical aniridia and foveal hypoplasia.	Likely pathogenic (PM2, PP1, PP2, PP3, PP4, PP5)
c.683‐3C>G	PMID: 32214788	Aniridia	A familial case of complete aniridia with nystagmus and cataract is reported.	Uncertain significance (PM2, PP3, PP1, PP5)
c.766‐3C>G	PMID: 26661695	Aniridia	Sporadic case is reported.	Likely pathogenic (PM2, PP3, PS2)
c.917‐9T>A	ClinVar: 372443	Not provided	Allele origin: germline	Uncertain significance (PM2, PP3, PP5)
c.917‐3C>G	ClinVar: 390567	Aniridia	Reported de novo status.	Likely pathogenic (PM2, PP3, PP5, PS2)
c.1032+3A>T	PMID: 25525159; Wildhardt, 1998	Aniridia	No additional information	Uncertain significance (PM2, PP3)
c.1033‐3C>G	ClinVar: 289617; PMID: 32360764	Aniridia	PMID 32360764 reported a case of partial aniridia, fine nystagmus and bilateral iris coloboma. Variant was observed de novo.	Likely pathogenic (PM2, PP3, PS2)
c.1183+4A>G	PMID: 9482572, 25,525,159	Aniridia	No additional information	Uncertain significance (PM2, PP3)
**Exonic SNVs**				
c.52G>A (p.G18R)	ClinVar: 1075401; LOVD: PAX6_000308; PMID: 12015275	Aniridia; Peters anomaly	Both sporadic and familial cases as well as de novo status are reported.	Pathogenic (PS1, PM1, PM2, PP3, PP5)
c.94C>G (p.L32V)	ClinVar: 800390; PMID: 18241071	Aniridia	Familial case is reported.	Likely pathogenic (PM1, PM2, PP3, PP5, PP1)
c.140A>C (p.Q47P)	ClinVar: 800400; PMID: 32360764	Aniridia	Familial case is reported.	Likely pathogenic (PM1, PM2, PM5, PP3, PP5, PP1)
c.141G>A (p.Q47Q)	PMID: 25678763	Aniridia?	Sporadic case is reported. Proband also has nystagmus, foveal hypoplasia, cataract, glaucoma and keratopathy.	Likely pathogenic (PM2, PP3, PS2)
c.155G>A (p.C52Y)	PMID: 25182519	Peters anomaly	Reported de novo status.	Likely pathogenic (PM1, PM2, PP3, PS2)
c.164A>C (p.K55T)	ClinVar: 430983; PMID: 28321846	Aniridia	Reported de novo status.	Likely pathogenic (PM1, PM2, PP3, PS2)
c.233T>G (p.V78G)	LOVD: PAX6_000828; PMID: 30167917, 33,594,928, 34,415,986	Aniridia; Foveal hypoplasia	PMID 30167917 reports a familial case of mild aniridia. The proband, referred for poor vision and nystagmus, showed slight corectopia and mild corneal neovascularisation upon detailed examination. Her mother and aunt exhibited similar findings, while her grandfather had advanced corneal vascularisation, opacity and foveal hypoplasia.	Likely pathogenic (PM1, PM2, PP3, PP1)
c.233T>C (p.V78A)	ClinVar: 283431; LOVD: PAX6_000856; PMID: 31700164; ISSN 1352‐240X	Aniridia; Foveal hypoplasia	PMID 31700164 reported a familial case of bilateral partial aniridia with foveal hypoplasia, nystagmus, optic nerve hypoplasia, strabismus and keratopathy	Likely pathogenic (PM1, PM2, PP3, PP1)
c.255C>T (p.S85S)	ClinVar: 418399; LOVD: PAX6_000761; PMID: 32360764	Aniridia	PMID 32360764 reported a case of nystagmus and partial aniridia.	Uncertain significance (PM2, PP3, PP5)
c.333C>A (p.V111V)	ClinVar: 800425; PMID: 10862096, 32,360,764	Aniridia	Reported de novo status.	Pathogenic (PS2, PS4, PM2, PP2, PP3, PP4)
c.485G>A (p.W162X)	ClinVar: 942257; PMID: 32214788	Aniridia	Familial case of complete aniridia with foveal hypoplasia, nystagmus and cataract is reported.	Pathogenic (PVS1, PM2, PP3, PP5)
c.681A>G (p.K227K)	ClinVar: 800448; PMID: 32360764	Aniridia	Reported de novo status.	Likely pathogenic (PM2, PP3, PP5, PS2)
c.682G>A (p.E228K)	ClinVar: 806642	Not provided	Allele origin: germline	Likely pathogenic (PM1, PM2, PM6, PP3, PS4)
c.763C>T (p.Q255X)	PMID: 11431688, 25,525,159, 34,101,622	Aniridia	PMID 34101622 reported a familial case of complete aniridia with foveal hypoplasia, nystagmus, glaucoma and cataract. Proband also has obesity, apnoea, asthma, hypertension, depression and irritable bowel syndrome.	Pathogenic (PVS1, PM2, PP3)
c.764A>G (p.Q255R)	PMID: 25678763	Aniridia	In reported familial case proband showed complete aniridia, but the proband's mother and son showed partial aniridia with the same variant.	Likely pathogenic (PM2, PP3, PM1, PP1)
c.765G>T (p.Q255H)	PMID: 10737978	Aniridia	Sporadic case is reported.	Likely pathogenic (PM2, PP3, PM1, PS2)
c.765G>C (p.Q255H)	ClinVar: 800452; PMID: 18241071, 32,360,764, 33,169,869	Aniridia	Both sporadic and familial cases, including de novo status, are reported. PMID 33169869 examines the effects of PAX6 mutations on glucose metabolism and insulin secretion and reports complete penetrance of the variant.	Pathogenic (PM1, PM2, PP3, PP5, PS2)
c.770G>A (p.W257X)	ClinVar: 800455; PMID: 18241071, 25,525,159, 32,360,764	Aniridia	PMID 18241071 reported sporadic cases of partial aniridia.	Pathogenic (PVS1, PM2, PP3, PP5)
c.1030C>T (p.Q344X)	PMID: 32467297	Aniridia	Sporadic case is reported.	Pathogenic (PVS1, PM2, PM6)
c.1183G>A (p.G395R)	PMID: 21850189, 25,525,159	Aniridia	Sporadic case is reported.	Likely pathogenic (PM1, PM2, PP3, PS2)

*Note:* Variants were named according to the reference NM_000280.4.

Next, a detailed analysis of exonic variants registered in the databases and predicted by SpliceAI as potentially influencing splicing was performed. All the exonic variants were initially classified as nonsense, missense and synonymous variants.

All five synonymous variants registered in the databases and predicted by SpliceAI as likely to affect splicing had a high DS (> 0.7). One of the five variants we have previously investigated experimentally [40], so the four remaining synonymous variants were selected for further functional study. A detailed analysis of these variants showed that two of them probably led to disruption of the donor splicing site, and two others may have led to the formation of a new donor splicing site in the corresponding exon.

Among the 15 missense variants registered in the databases and predicted by SpliceAI to be likely to affect splicing, three were confirmed by functional studies, so 12 missense variants were selected for this study, among which six variants had a high DS (> 0.7). A detailed analysis of these variants showed that six of them probably led to the disruption of the donor splice site, five to the formation of a new donor splice site and one variant to the formation of a new acceptor splicing site.

Since the mechanism of pathogenicity of nonsense variants is the most obvious, all 16 nonsense variants registered in the databases and predicted by SpliceAI as likely to affect splicing were further analysed to assess whether the stop codon generated as a result of the mutation remained in the aberrantly spliced transcript. In addition, among all nonsense variants, we searched for variants that can lead to in‐frame deletions, leading to a milder phenotype than expected in the case of a nonsense mutation. As a result, three nonsense variants were selected for this study, which probably led to the disruption of the wild‐type splicing donor site, the formation of a new splicing donor site in the exon and the formation of a new splicing acceptor site. In addition, we included one artificial nonsense variant (c.485G>A) that has not been reported in the databases. Our *in silico* predictions suggest that this variant may create a new donor splicing site in exon 7, leading to an in‐frame mRNA truncation. Furthermore, this splicing change would remove the initial stop codon from the mRNA, causing the ‘nonsense’ variant to actually function as an in‐frame deletion.

In total, 20 exonic *PAX6* variants were selected for the experimental study, including 4 synonymous, 12 missense and 4 nonsense variants (Table 1). Most of these variants (15/20, 75%) had a SpliceAI delta score > 0.2, of which six variants had a delta score > 0.5 (recommended/pathogenic) and six variants had a delta score > 0.8 (high precision/pathogenic).

**FIGURE 2:**
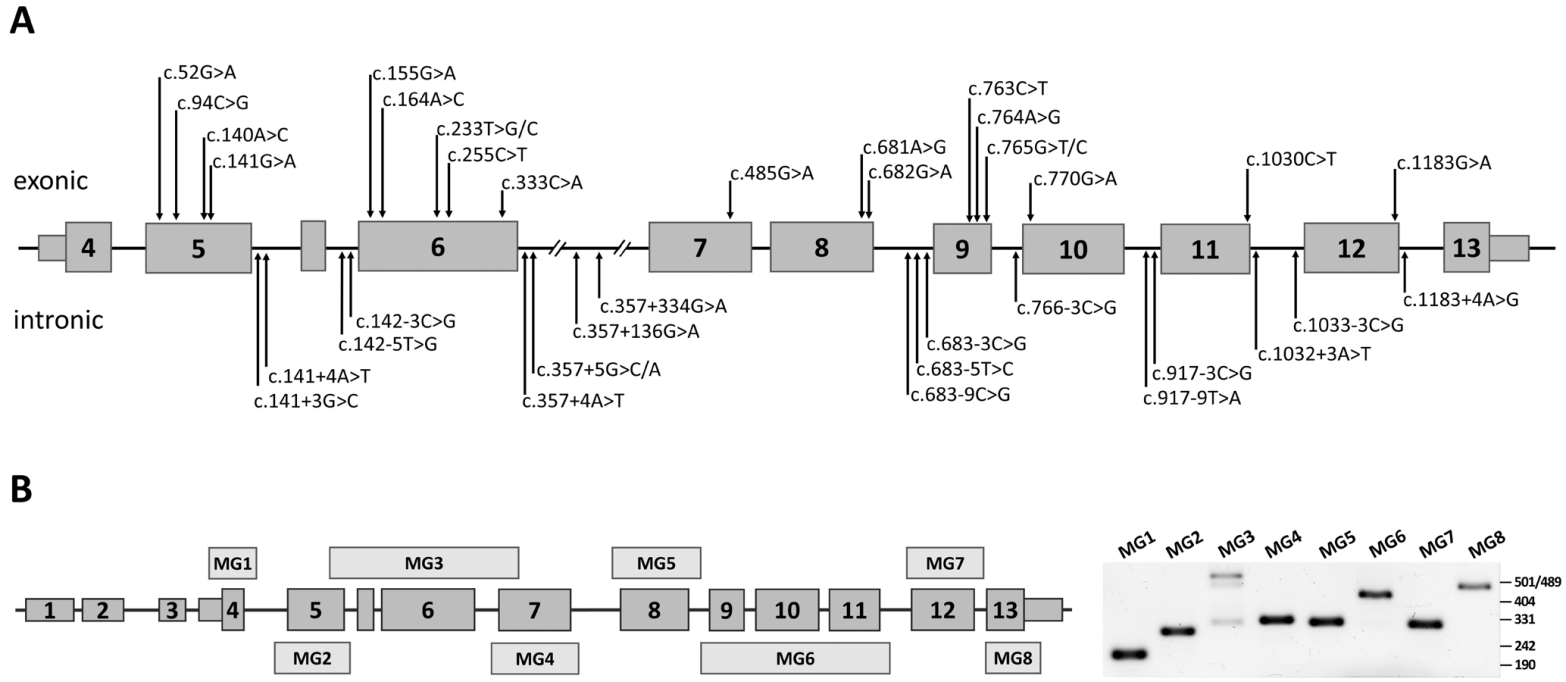
A. The schematic diagram of the *PAX6* gene coding sequence and the localisation of intronic and exonic variants selected for minigene splicing assay. B. Left: Mapping of the designed minigene constructions onto the scheme of the *PAX6* gene. Right: Electrophoresis of RT‐PCR products derived from HEK293 cells transfected with wild‐type minigenes.

Thus, we selected 18 intronic and 20 exonic SNVs in the *PAX6* gene for further functional assessment of their impact on splicing (Figure 2A).

### 3.2 Development of a Minigene Expression System for the Functional Analysis of *PAX6* Splicing Variants

To experimentally evaluate the effects of the selected variants on pre‐mRNA splicing, a minigene expression system was developed. To cover the entire coding sequence of *PAX6*, we divided it into eight loci and designed eight corresponding minigene constructs. Seven of the eight constructs contained one exon of the *PAX6* gene, while one construct included closely spaced exons 9, 10 and 11 (Figure 2B).

The *PAX6* gene regions corresponding to the designated constructs were amplified from control human gDNA and inserted into the pSpl3‐Flu2‐TK‐del plasmid vector. The nucleotide sequences of all the plasmids were confirmed by Sanger sequencing.

To confirm correct splicing, the created minigene constructions were transfected into HEK293T cells. Total RNA was isolated 48 h after transfection, and RT‐PCR analysis was performed using primers specific for plasmid exons. The correspondence of the PCR product to a normally spliced transcript was established using agarose or polyacrylamide gel electrophoresis and fragment analysis and confirmed by Sanger sequencing and targeted NGS (Figure S1).

Five of the eight created wild‐type constructions [containing exons 4 (MG1), 7 (MG4), 8 (MG5), 12 (MG7) and 13 (MG8)] led to the production of normally spliced transcripts containing target exons. However, three constructions expressing exons 5 (MG2), 6 (MG3) and exons 9 to 11 (MG6) exhibited some unexpected splicing events.

**FIGURE 3:**
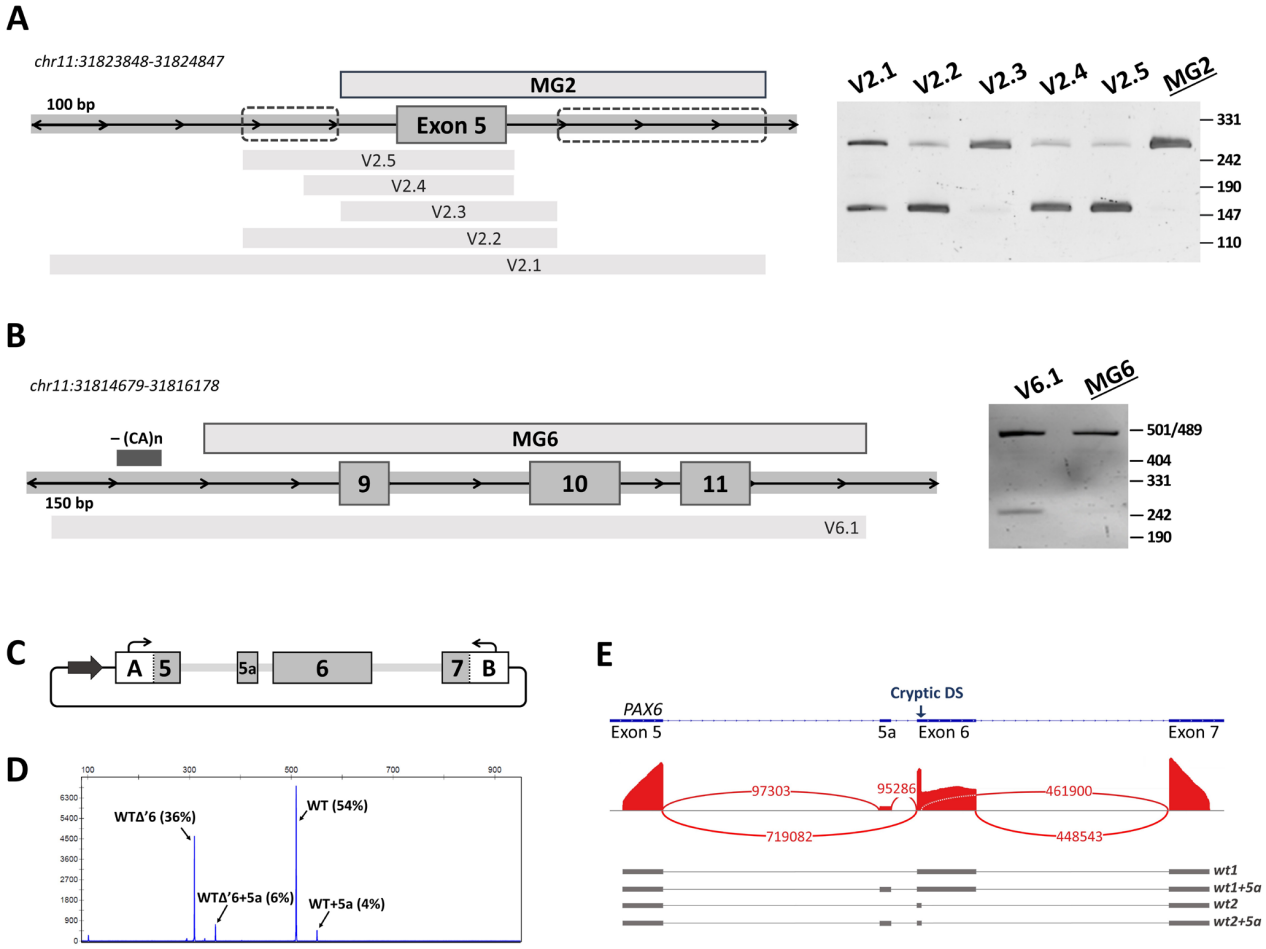
Development of a minigene expression system for exons with unexpected splicing events (exon 5 (A), exons 9–10 (B) and exon 6 (C, D, E)). A. Left: The *PAX6* gene locus containing exon 5 and a schematic representation of all minigene construction we created to achieve correct exon inclusion. The dotted lines indicate putative locations of splicing regulatory elements. The final variant with correct exon 5 inclusion is labelled MG2. Right: Electrophoresis of RT‐PCR products derived from HEK293 cells transfected with all variants of exon 5 minigene. B. Left: The *PAX6* gene locus containing exons 9, 10, 11 and a schematic representation of two variants of the minigene construction. The dark rectangle indicates a simple repeat in intron 8. The final variant is labelled MG6. Right: Electrophoresis of RT‐PCR products derived from HEK293 cells transfected with two variants of exons 9‐10‐11 minigene. C‐E. Alternative splicing in a minigene construction expressing exon 6. C. Schematic of a minigene plasmid expressing exon 6 of the *PAX6* gene. In this construction, adjacent exons 5 and 7 were partially fused to the surrounding plasmid exons. D. Fragment analysis of RT‐PCR products demonstrates the observed ratio of wild‐type isoforms. E. Targeted next‐generation sequencing of RT‐PCR product. Sashimi plot visualises splice junctions. Below is a schematic representation of the observed isoforms.

In the case of exon 5 construction, we observed skipping of the target exon along with the correctly spliced isoform. A region of the *PAX6* gene, cloned into a plasmid vector to create the exon 5 minigene, included the 410‐bp‐long flanking sequences of intron 4 and 310 bp of intron 5 (V2.1 in Figure 3A). We hypothesised that the flanking intronic sequences contain some regulatory elements that may prevent correct splicing by being removed from their full‐genome context. Hence, a new construction containing a shortened intron sequence (182 bp of intron 4 and 63 bp of intron 5) was created (V2.2 on Figure 3A). However, testing on HEK293T cells showed that the shortening of the flanking intronic sequences resulted in complete skipping of exon 5.

To solve the problem of exon 5 skipping, three new minigene constructs were created. Among them, one had a minimal flanking sequence of intron 4, which corresponds to one of the bioinformatically predicted branch points located at Position −52 (construction V2.3). The other included a bit longer sequence of intron 4, corresponding to the strongest predicted branching point at Position −89, but the flanking sequence of intron 5 was shortened to a minimum of 11 bp (construction V2.4). The third one also had a minimal intron 5 but retained all 182 bp of flanking sequence of the intron 4 (constructions V2.5). Subsequent testing showed that the highest level of exon 5 inclusion was observed in construction V2.3. However, a small amount of the isoform with exon 5 skipping was still observed by electrophoresis. After analysing all the obtained data, we hypothesised the presence of two types of regulatory elements in the area surrounding exon 5: a splicing suppressor in intron 4 and a splicing enhancer in intron 5 (Figure 3A). Given this hypothesis, we extended the inserted intronic sequence in the V2.3 construction so that it included the putative splicing enhancer. Transfection of the resulting plasmid followed by RT‐PCR analysis and Sanger sequencing confirmed the correct splicing. Thus, a minigene construction of exon 5 (MG2) was successfully obtained.

The MG6 construction included exons 9, 10 and 11 with separating introns 9 and 10 and flanking sequences (470 bp of intron 8 and 195 bp of intron 11). RT‐PCR analysis of samples obtained after transfection revealed a minor isoform with skipping of exons 10 and 11 in addition to the correctly spliced transcript (Figure 3B, V6.1). Detailed analysis of the cloned genomic region revealed that the sequence of intron 8 includes a simple repeat, usually containing several regulatory elements that may affect splicing efficiency. So, to avoid the multiple exons skipping, we created a new construction with a shortened sequence of intron 8 (220 bp). Testing of the new construction did not reveal any incorrectly spliced isoforms in the RT‐PCR product (Figure 3B). So, a minigene construction expressing exons 9, 10 and 11 (MG6) was also successfully obtained.

A feature of the construction expressing exon 6 is that adjacent exons 5 and 7 of the *PAX6* gene were partially fused with plasmid exons expressing RFP and GFP, respectively (Figure 3C). The known difficulty in alternative splicing of exon 6 is due to the presence of a cryptic donor site at Position c.157 and alternative exon 5a. This leads to the shortening of exon 6 to 15 bp (WT∆’6) and the inclusion of the alternative exon 5a (WT5a), respectively [48, 49]. Fragment analysis of the PCR product obtained after transfection of the WT minigene showed the presence of a mixture of transcripts: WT, WT + 5a, WT∆‘6 and WT∆’6 + 5a, which was also confirmed by targeted next‐generation sequencing (Figure 3D,E).

Thus, eight minigene constructions expressing all coding exons of the *PAX6* gene were obtained and successfully tested in a model cell line (Figure 2B). All target exons are well expressed and normally spliced, and all constructs are suitable for functional analysis of the potentially splice‐affecting variants in the *PAX6* gene.

### 3.3 Functional Analysis of the *PAX6* Intronic Variants

Eighteen intronic *PAX6* variants located outside the canonical splice site dinucleotides were selected for functional analysis (Table 1). Half of these variants (9 of 18) were located at different positions in the acceptor splicing sites of *PAX6*. Of the remaining nine variants, seven were located in donor splicing sites and two variants were deep intronic.

Functional analysis of the intronic variants using minigene construction revealed a range of different splicing impairments (Table 2, Figure S2).

**TABLE 2:** Functional analysis of the intronic and exonic SNVs in the *PAX6* gene.

Variant	SpliceAI		Minigene assay outcome			Reclassification*
	Prediction	DS	Aberrant transcripts	Predicted protein change	WT	
**Intronic SNVs**						
c.141+3G>C	New 5'SS	DG = 0.37	Skipping exon 5 (30%) 73‐nt intron retention (4%)	p.Ser4Asnfs*22 p.Ser49Ilefs*31	66%	VUS
c.141+4A>T	New 5'SS	DG = 0.41	Skipping exon 5 (60%) 73‐nt intron retention (10%)	p.Ser4Asnfs*22 p.Ser49Ilefs*31	30%	LP → P
c.142‐5T>G	New 3'SS	AG = 0.98	Skipping exon 6 (33%) 4‐nt intron retention (55%)	p.Asn50_Ser121del p.Val48Alafs*9	12%	LP **→** P
c.142‐3C>G	New 3'SS	AG = 0.2	Skipping exon 6 (100%)	p.Asn50_Ser121del	ND	VUS **→** LP
c.357+4A>T	New 5'SS	DG = 0.47	Skipping exon 6 (44%) 108‐nt truncation (17%)	p.Asn50_Ser121del p.Val84_Ser119del	39%	LP **→** P
c.357+5G>A	5'SS broken New 5'SS	DL = 0.84 DG = 0.55	Skipping exon 6 (40%) 108‐nt truncation (17%)	p.Asn50_Ser121del p.Val84_Ser119del	34%	LP **→** P
c.357+5G>C	5'SS broken New 5'SS	DL = 0.94 DG = 0.51	Skipping exon 6 (51%) 108‐nt truncation (15%)	p.Asn50_Ser121del p.Val84_Ser119del	34%	LP **→** P
c.357+136G>A	New 3'SS	AG = 0.39	194‐nt PE inclusion (22%)	p.Val120Glyfs*48	78%	LP
c.357+334G>A	New 5'SS	DG = 0.64	194‐nt PE inclusion (18%) 13‐nt PE inclusion (5%) Intron retention (4%)	p.Val120Glyfs*48 p.Val120Asnfs*17 p.Ile123Leufs*18	73%	VUS
c.683‐9C>G	3'SS broken	AL = 0.80	Skipping exon 9 (83%) 8‐nt intron retention (17%)	p.Glu228Glyfs*5 p.Glu228Valfs*19	ND	LP **→** P
c.683‐5T>C	3'SS broken	AL = 0.63	Skipping exon 9 (100%)	p.Glu228Glyfs*5	ND	LP **→** P
c.683‐3C>G	3'SS broken	AL = 0.86	Skipping exon 9 (100%)	p.Glu228Glyfs*5	ND	VUS **→** LP
c.766‐3C>G	3'SS broken New 3'SS	AL = 0.45 AG = 0.55	Skipping exons 9–11 (5%) Skipping exons 10–11 (41%) 20‐nt truncation (48%)	p.Glu228Alafs*26 p.Val256_Gln344del p.Val256Glyfs*21	6%	LP **→** P
c.917‐9T>A	3'SS broken New 3'SS	AL = 0.92 AG = 0.98	Skipping exons 10–11 (16%) Skipping exon 11 (34%) 7‐nt intron retention (50%)	p.Val256_Gln344del p.Val306Alafs*26 p.Val306Alafs*37	ND	VUS **→** LP
c.917‐3C>G	3'SS broken	AL = 0.17	Skipping exons 10–11 (23%) Skipping exon 11 (77%)	p.Val256_Gln344del p.Val306Alafs*26	ND	LP **→** P
c.1032+3A>T	5'SS broken	DL = 0.34	Skipping exons 10–11 (13%) Skipping exon 11 (87%)	p.Val256_Gln344del p.Val306Alafs*26	ND	VUS **→** LP
c.1033‐3C>G	3'SS broken New 3'SS	AL = 0.98 AG = 0.99	2‐nt intron retention (100%)	p.Pro345Serfs*21	ND	LP **→** P
c.1183+4A>G	5'SS broken	DL = 0.17	Skipping exon 12 (82%)	p.Pro345Aspfs*130	18%	VUS **→** LP
**Exonic SNVs**						
c.52G>A (p.G18R)	New 3'SS	AG = 0.48	ND	ND	100%	P
c.94C>G (p.L32V)	New 5'SS	DG = 0.79	ND	ND	100%	LP
c.140A>C (p.Q47P)	5'SS broken	DL = 0.29	Skipping exon 5 (85%) 73‐nt intron retention (12%)	p.Ser4Asnfs*22 p.Ser49Ilefs*31	3%	LP **→** P
c.141G>A (p.Q47Q)	5'SS broken New 5'SS	DL = 0.73 DG = 0.31	Skipping exon 5 (35%) 73‐nt intron retention (65%)	p.Ser4Asnfs*22 p.Ser49Ilefs*31	ND	LP **→** P
c.155G>A (p.C52Y)	New 5'SS	DG = 0.13	ND	ND	100%	LP
c.164A>C (p.K55T)	New 5'SS	DG = 0.13	ND	ND	100%	LP
c.233T>G (p.V78G)	New 5'SS	DG = 0.17	108‐nt truncation (8%)	p.Val84_Ser119del	92%	LP
c.233T>C (p.V78A)	New 5'SS	DG = 0.16	108‐nt truncation (2%)	p.Val84_Ser119del	98%	LP
c.255C>T (p.S85S)	New 5'SS	DG = 0.78	108‐nt truncation (62%)	p.Val84_Ser119del	38%	VUS‐ > LP
c.333C>A (p.V111V)	New 5'SS	DG = 0.94	27‐nt truncation (36%)	p.Cys112_Val120del	64%	P
c.485G>A (p.W162X)	New 5'SS	DG = 0.59	ND	ND	100%	P
c.681A>G (p.K227K)	5'SS broken New 5'SS	DL = 0.81 DG = 0.53	39‐nt intron retention (65%) Full intron retention (35%)	p.Glu228delins14 p.Glu228_Gln422delinsTer23	ND	LP **→** P
c.682G>A (p.E228K)	5'SS broken New 5'SS	DL = 0.93 DG = 0.56	39‐nt intron retention (70%) Full intron retention (30%)	p.Glu228delins14 p.Glu228_Gln422delinsTer23	ND	LP **→** P
c.763C>T (p.Q255X)	5'SS broken	DL = 0.58	Skipping exon 9 (100%)	p.Glu228Glyfs*5	ND	P
c.764A>G (p.Q255R)	5'SS broken	DL = 0.70	Skipping exon 9 (100%)	p.Glu228Glyfs*5	ND	LP **→** P
c.765G>T (p.Q255H)	5'SS broken	DL = 0.93	Skipping exon 9 (100%)	p.Glu228Glyfs*5	ND	LP **→** P
c.765G>C (p.Q255H)	5'SS broken	DL = 0.92	Skipping exon 9 (100%)	p.Glu228Glyfs*5	ND	P
c.770G>A (p.W257X)	New 3'SS	AG = 0.17	Skipping exons 10–11 (18%)	p.Val256_Gln344del	82%	P
c.1030C>T (p.Q344X)	New 5'SS	DG = 0.99	Skipping exons 10–11 (6%) Skipping exon 11 (8%) 4‐nt truncation (81%)	p.Val256_Gln344del p.Val306Alafs*26 p.Met343Ilefs*21	5%	P
c.1183G>A (p.G395R)	5'SS broken	DL = 0.34	Skipping exon 12 (100%)	p.Pro345Aspfs*130	ND	LP **→** P

*Note:* Variants were named following Human Genome Variation Society (HGVS) guidelines with references NM_000280.4 and NP_000271.1. *The reclassification made by applying the PS3 criteria.

Abbreviations: AG, acceptor gain; AL, acceptor loss; DG, donor gain; DL, donor loss; DS, delta score; LP, likely pathogenic; ND, not detected; nt, nucleotide; P, pathogenic; VUS, variant of uncertain significance; SS, splice site; WT, wild‐type.

**FIGURE 4:**
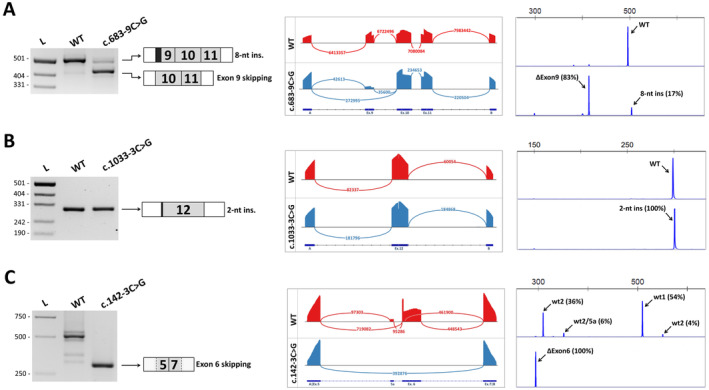
Examples of functional analysis of *PAX6* intronic variants. A. The c.683‐9C>G variant leads to the out‐of‐frame skipping of exon 9 and retention of 8 nucleotides of intron 8. B. The c.1033‐3C>G variant leads to the out‐of‐frame retention of 2 nucleotides of intron 12. C. The c.142‐3C>G variant leads to the total skipping of both major isoforms of exon 6. Figures A‐C show from left to right: Electrophoresis of RT‐PCR products obtained from transfected HEK293 cells and schematic of the observed splicing events; targeted next‐generation sequencing of RT‐PCR products. Sashimi plot visualises splice junctions; fragment analysis of RT‐PCR products demonstrates the observed isoform ratio.

According to SpliceAI, four of nine variants located in the 3′ splicing site (c.683‐3C>G, c.683‐5 T>C, c.683‐9C>G and c.917‐3C>G) likely lead to the loss of the acceptor splicing site. Functional analysis revealed that all of these variants led to skipping of the corresponding exons followed by frameshift. The c.683‐9C>G variant, in addition to skipping exon 9, also leads to the formation of a new weak acceptor site followed by out‐of‐frame 8‐nt intron retention (approximately 17% of the total number of transcripts) (Figure 4A). This event was also predicted by SpliceAI, but was rated as low probability (DS_AG = 0.16).

For another three variants located in the 3′ splicing site (c.766‐3C>G, c.917‐9 T>A and c.1033‐3C>G), SpliceAI predicted an approximately equal probability of both the formation of a new site and the destruction of an existing acceptor site. Functional analysis revealed that variants c.766‐3C>G and c.917‐9 T>A indeed lead to the appearance of several aberrant transcripts containing both isoforms formed as a result of the formation of a new acceptor site and isoforms with exon skipping due to the destruction of the wild‐type site. The c.1033‐3C>G variant showed only one mis‐spliced transcript retaining 2 bp of the intron due to the creation of a new acceptor site (Figure 4B).

For the remaining two variants located in the 3′ splicing site (c.142‐3C>G and c.142‐5T>G), the most likely consequence was the appearance of the new acceptor site (AG). Functional analysis confirmed that the c.142‐5T>G variant resulted in the creation of a new acceptor site followed by 4 bp intron retention. This variant also led to partial exon 6 skipping. The c.142‐3C>G variant was predicted by SpliceAI to activate a cryptic acceptor site located 38 bp further in the exon (DS_AG = 0.19), and, unexpectedly, also affects the previously mentioned cryptic donor site at Position c.157 of exon 6 (DS_DL = 0.30). Functional analysis revealed complete exon 6 skipping in this case (Figure 4C). However, this observation is in good agreement with the MaxEntScan assessment, which shows that the c.142‐3C>G variant dramatically reduces the acceptor site strength (from 11.06 to −2.47).

SpliceAI predicted that two of the seven variants located at 5′ splice sites (c.357+5G>C and c.357+5G>A) were likely to disrupt splice sites but were also likely to create a new donor site. Both variants showed the skipping of the corresponding exon as the main splicing event and also resulted in the activation of the cryptic splicing site in exon 6 followed by a 108‐nt truncation. Similar cases of activation of cryptic sites due to pathogenic variants in the exon 6 donor splicing site have been previously described in the literature [49]. In addition, in both cases, significant (> 30%) residual expression of the wild‐type transcript was observed.

Next, two variants located in 5′ splicing sites (c.1032+3A>T and c.1183+4A>G) were predicted by SpliceAI only to destroy the donor site, and in both cases, exon skipping was the main splicing event detected. The c.1183+4A>G variant led to significant skipping of exon 12 (83%) despite a low SpliceAI delta score (DS_DL = 0.17). According to the MAXENT assessment, this variant reduces the strength of the wild‐type donor site only from 9.60 to 8.07.

For the remaining three variants located in 5′ splicing sites (c.141+3G>C, c.141+4A>T and c.357+4A>T), SpliceAI predicted the formation of a new donor site. For variants c.141+3G>C and c.141+4A>T, a new site was predicted at 74 and 73 nucleotides upstream in intron 5, respectively. Functional analysis revealed that the use of this new site resulted in only a small amount of aberrant transcripts with 75‐nt intron retention (5% and 10%, respectively). Both of these variants also attenuate the wild‐type donor site (MAXENT: from 4.28 to 2.07 and 1.16, respectively), resulting in exon 5 skipping (30% and 60%, respectively). The c.357+4A>T variant led to exon 6 skipping, along with activation of the cryptic donor site in exon 6, followed by a 108‐nt truncation, similar to the above‐described variants in intron 6.

Finally, two variants were located deep in intron 6 of the *PAX6* gene. According to the SpliceAI prediction, these variants should lead to the formation of new acceptor (c.357+136G>A) and donor (c.357+334G>A) splicing sites. Functional analysis revealed that both of these variants led to the inclusion of different levels of the 194 bp pseudoexon. Analysis of the nucleotide sequence of intron 6 revealed cryptic acceptor and donor splicing sites located at Positions c.357+138 and c.357+331, respectively. These variants increase the strength of the cryptic sites, which leads to pseudoexon inclusion. The c.357+334G>A variant also leads to a slight (~5%) incorporation of a small part of the mentioned pseudoexon (13 bp) corresponding to the use of a different cryptic acceptor site at Position c.357+319.

Thus, all 18 tested intronic variants in the *PAX6* gene were shown to disrupt normal pre‐mRNA splicing. In most cases (15/18), the intronic variant resulted in complete or partial skipping of the corresponding exon. It should be noted that in 10/18 cases, along with mis‐spliced transcripts, we observed a variable amount (77%–6%) of the wild‐type transcript, which should probably lead to a milder phenotype. A deeper analysis of the clinical data is required to confirm this hypothesis.

### 3.4 Functional Analysis of the *PAX6* Exonic Variants

Twenty exonic variants in the *PAX6* gene were selected for functional analysis, including 4 synonymous, 12 missense and 4 nonsense variants (Table 1).

According to SpliceAI prediction, two of four selected synonymous variants (c.255C>T and c.333C>A) should lead to new donor splicing site creation. Functional analysis confirmed that in both cases, the variants strengthen the cryptic donor site in exon 6, followed by in‐frame deletions of 108 bp and 27 bp, respectively (Table 2). It should be noted that these variants are located in the previously described cryptic splicing sites of exon 6 [49].

**FIGURE 5:**
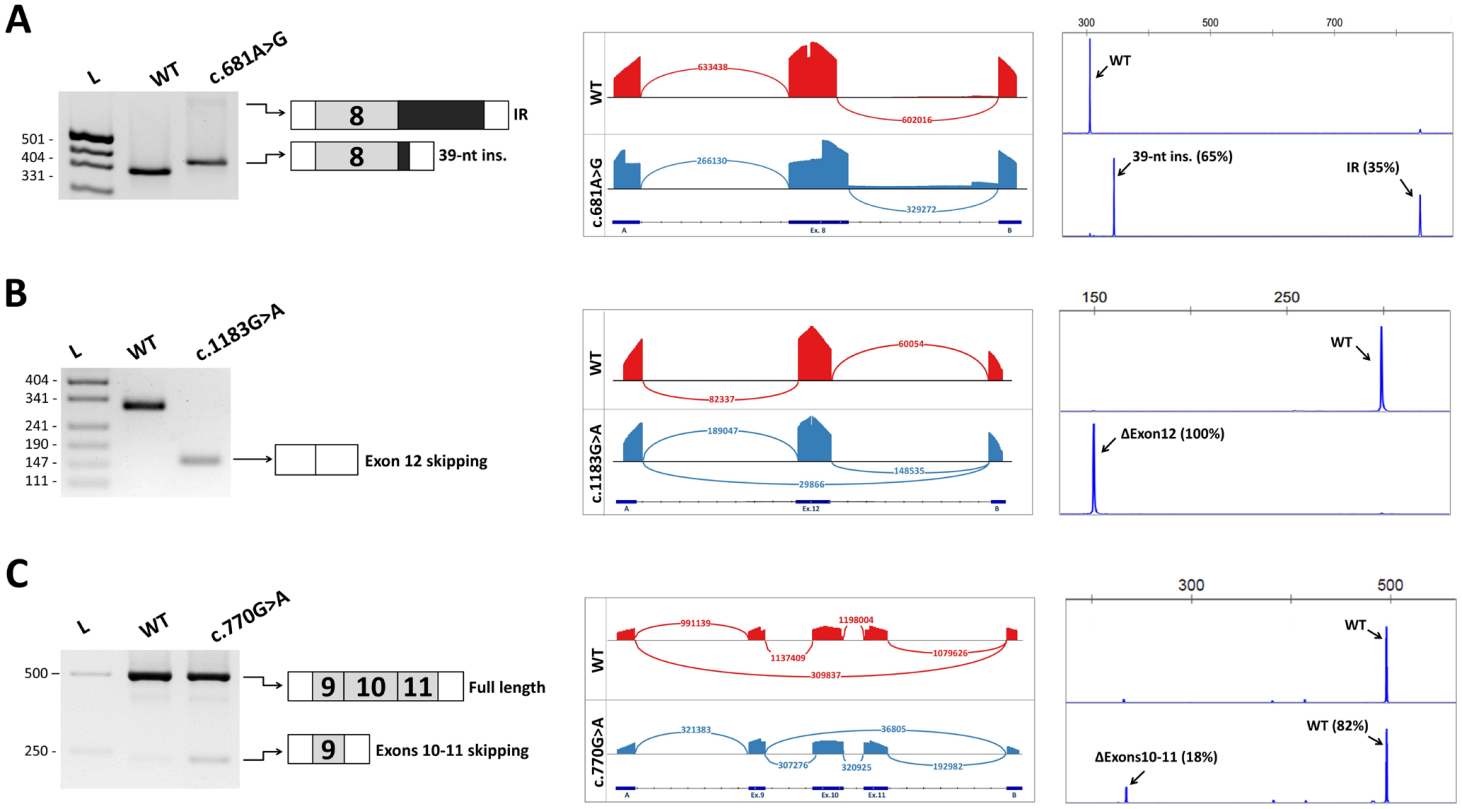
Examples of functional analysis of *PAX6* intronic variants. A. Synonymous variant c.681A>G leads to partial and complete retention of intron 8. B. Missense variant c.1183G>A leads to complete skipping of exon 12. C. Nonsense variant c.770G>A was predicted to lead to activation of the weak cryptic acceptor site, but the main isoform remains a full‐length transcript. Figure 5A–C shows from left to right: Electrophoresis of RT‐PCR products obtained from transfected HEK293 cells and a schematic of the observed splicing events; targeted next‐generation sequencing of RT‐PCR products. Sashimi plot visualises splice junctions; fragment analysis of RT‐PCR products demonstrates the observed isoform ratio.

Two remain synonymous variants, located in the last (c.141G>A) and penultimate (c.681A>G) nucleotides of the exon and, according to SpliceAI, could lead to the loss of the canonical site and the activation of the cryptic or formation of a new donor site. Functional analysis showed that in both cases the main consequence was the activation of the cryptic donor site in the upstream intronic sequence and the insertion of 75 or 39 bp, respectively. The c.141G>A variant also resulted in the skipping of exon 5 (p.Ser49Ilefs*31). Notably, in the case of the 681A>G variant, the insert 39 bp does not contain a premature stop codon and should result in 13 amino acid insertions (Figure 5A). However, this insertion disrupts the structure of the *PAX6* homeodomain, which is likely to affect its function. According to the MAXENT assessment, the cryptic splicing site leading to the 39 bp insertion is not very strong (4.06) and is probably not used by the spliceosome as the main site. Therefore, in addition to the 39 bp insertion, we observed that some (35%) of the isoforms retained the entire sequence of intron 8, which we cloned into the expression plasmid. Analysis of this sequence showed that it could lead to the insertion of 23 amino acids followed by a stop codon. As a result, the *PAX6* protein does not contain 172 C‐terminal amino acids, including most of the homeodomain.

Functional analysis revealed that of the 12 missense variants selected for the study, 6 did not affect splicing. Four of them were located in exon 6 (c.155G>A, c.164A>C, c.233T>G, c.233T>C) and had a low DS (< 0.2). Variants c.52G>A and c.94C>G located in exon 5, despite relatively high DS, were also not found to be splice‐affecting. It is notable that all six variants are concentrated in the paired domain sequences, which contain most of the reported *PAX6* missense variants.

According to SpliceAI, the six missense variants should lead to the loss of the donor splicing site. For most of these variants (c.140A>C, c.764A>G, c.765G>C, c.765G>T, c.1183G>A), the main observed effect was out‐of‐frame skipping of the corresponding exon (Figure 5B). In the c.140A>C variant, in addition to exon 5 skipping, we observed activation of the cryptic donor splicing site and retention of 75 bp of intron 5 followed by a frameshift. The last variant, c.682G>A, located in the last nucleotide of exon 8, led to the same effects as the synonymous variant c.681A>G described above.

Functional analysis of nonsense variants revealed that three of the four tested variants affected splicing. The hypothetical c.485G>A variant predicted *in silico* showed no effect on splicing, despite a high DS (0.59). Based on the SpliceAI prediction, the remaining 3 variants are expected to have different outcomes. The c.763C>T variant was predicted to destroy the donor splicing site in exon 9. Functional analysis confirmed the loss of the donor site and exon 9 skipping followed by a frameshift. The c.1030C>T variant, predicted as a donor gain, indeed leads to the formation of a new donor splicing site according to the results of the functional analysis. This results in the deletion of the 4 bp region of exon 11. The variant c.770G>A, according to expectations, should lead to the activation of the cryptic acceptor site in exon 10. However, functional analysis showed that a weak acceptor site is formed, and the proportion of such transcripts (with 14‐nt truncation) is only 2% of the total number. The main isoform observed in this case was the wild type (Figure 5C).

Thus, of the 20 exonic variants tested, 4 synonymous, 6 missense and 3 nonsense variants affected pre‐mRNA splicing. Moreover, in seven patients, the main consequence of splicing failure was a frameshift, essentially resulting in loss‐of‐function variants of these SNVs (Table 2, Figure S2).

## 4 Discussion

Recent advancements in next‐generation sequencing (NGS) have significantly enhanced the identification of variants in the *PAX6* gene, which is associated with aniridia and other ocular disorders. NGS has improved diagnostic yield, allowing for the detection of pathogenic *PAX6* variants in patients with atypical presentations [50]. However, as the number of variants identified rises, so does the number of variants that are challenging to interpret. This is particularly true for noncanonical splice site variants. The consequences of incorrect splicing cannot always be predicted bioinformatically, and functional studies are required to accurately determine their pathogenicity. As such studies are not routine, the percentage of pathogenic splicing variants is likely underestimated by current diagnostic methods [51]. For example, of the 149 *PAX6* single nucleotide variants registered as Pathogenic in the ClinVar database to date (July 2024), 48 SNVs (~33%) are intronic. Furthermore, 42 of the 48 pathogenic intronic SNVs (~90%) are located in the terminal AG‐GT dinucleotides of the splice sites. On the other hand, 23 of 144 (16%) *PAX6* SNVs reported in ClinVar as variants of uncertain significance (VUS) are noncanonical splice sites or deep intronic variants. These observations warrant further investigation and reassessment of variants classified as uncertain significance.

Recent studies have highlighted the complexity of *PAX6* gene splicing, particularly in exon 6, and its role in congenital aniridia. Multiple variants, including deep intronic, synonymous and noncanonical splice site mutations, can disrupt normal splicing patterns [40, 49, 52]. However, these studies have investigated patient‐specific variants, and the landscape of pathogenic splicing variants in the *PAX6* gene remains incompletely understood. The proportion of *PAX6* variants whose influence on splicing has been experimentally confirmed remains small.

To bridge this gap, we designed and tested a system of eight minigene constructions to experimentally investigate the effect of *PAX6* variants on splicing. This approach has previously been used to functionally characterise both canonical splice site variants and deep intronic variants, as well as variants in coding and noncoding regions of the *PAX6* gene [35, 53, 54]. Our system for the first time includes constructions expressing all coding exons of the *PAX6* gene and their surrounding intronic regions, allowing functional characterisation of any exon and a significant proportion of intronic variants potentially affecting splicing.

To test and validate our system, we performed functional analysis of 18 intronic and 20 exonic potentially spliceogenic variants reported in databases but not experimentally confirmed. We demonstrated that 13 of 20 exonic and all 18 intronic variants affected splicing. Notably, approximately 60% of the variants studied resulted in two or more splicing events. We used a comprehensive approach, including both regular electrophoresis and Sanger sequencing, as well as fragment analysis and deep target sequencing, which allowed us to clarify all cases that were difficult to interpret. These are mainly variants in the locus surrounding exon 6, which is known for complex and understudied splicing patterns. Notably, in the course of our work, several variants at this locus were investigated. Thus, Tarilonte et al. analysing the c.333C>A synonymous variant using a minigene splicing assay also revealed activation of the cryptic donor site and a 27‐nt shortening of exon 6 [49]. In turn, Tamayo et al. analysed c.357+136 and c.357+334 deep intronic variants using minigene splicing assays and nanopore‐based long‐read sequencing [52]. We observed similar splicing patterns using differently designed minigene constructions and approaches for analysis, which suggests that our data are highly reliable.

Eighteen intronic *PAX6* variants located outside the canonical ± 1.2 dinucleotides were selected for the study, including two deep intronic variants. Although such variants can be located in noncanonical but significant splicing site nucleotides, they are usually annotated as ‘intronic’ rather than ‘splicing’ which may complicate the assessment of their pathogenicity. For instance, in a study focused on developmental disorders, Lord et al. estimated that 35%–40% of pathogenic variants in noncanonical splice site positions may be missing from public databases [55]. On the other hand, we found 33 intronic variants in the most commonly used clinical databases, which were not assessed by SpliceAI as being potentially splice‐altering. An analysis of the sources revealed that only 2 of the 33 variants have been experimentally confirmed to affect splicing, while all others are likely to be nonpathogenic. Our functional analysis revealed that all 18 selected variants disrupted normal splicing and resulted in a range of aberrant transcripts. These results confirm the efficacy of the developed minigene expression system and emphasise the necessity of accurate prediction, interpretation and experimental validation of noncanonical splicing variants.

SpliceAI identified 36 of 249 exonic variants in the *PAX6* gene as potentially splicing disruptive. Firstly, we focused on four synonymous variants previously not characterised functionally. In recent years, there has been significant progress in understanding the molecular mechanisms whereby synonymous substitutions affect protein function and disease pathogenesis. These include altering mRNA stability, translation rates and protein folding [56, 57]. Disruption of splicing is one of the major mechanisms of pathogenicity of synonymous variants, and it is estimated that up to 45% of synonymous variants can alter pre‐mRNA splicing [58]. Our functional analysis showed that all four synonymous variants tested lead to splicing disruption, which fundamentally shifts the assessment of their pathogenicity. On the other hand, 21 of the 26 synonymous variants found in the databases were not considered by SpliceAI as potentially spliceogenic. However, a more detailed analysis showed that only one of these variants was interpreted as a VUS, whereas most variants were benign or probably benign. Moreover, 10 of the 21 variants had population frequencies.

In contrast to synonymous variants, of the 131 missense variants not predicted by SpliceAI, only 9 were annotated as VUS, whereas the majority were likely pathogenic or pathogenic. Our functional analysis showed that 6 of the 12 selected missense variants actually disrupt pre‐mRNA splicing, with variants c.140A>C, c.764A>G, c.765G>C, c.765G>T and c.1183G>A producing frameshift or PTC‐containing transcript as the main mis‐spliced isoform. Thus, the main mechanism of their pathogenicity is related to effects on splicing, whereas the remaining six variants (two of which had low DS) are probably ‘true missense’ that are pathogenic through amino acid changes. Notably, all six missense variants that have not demonstrated any splicing effects are located in the paired domain, as are most of the described missense variants of *PAX6* [30, 31].

Nonsense variants can occasionally influence splicing patterns by altering regulatory or consensus sequences or by creating new splicing sites [59, 60, 61]. If this alternative splicing maintains a reading frame, it may yield a semi‐functional protein expression. Haque et al. term this mechanism ‘manufactured splice rescue’, predicting that about 23% of nonsense variants cause in‐frame deletions, constituting < 10% of the coding transcript [62]. In this work, we functionally characterised 4 *PAX6* variants, finding that 3 affected splicing. The c.770G>A variant led to about 17% aberrant transcripts lacking stop codons, with the full‐length isoform at roughly 78%. In contrast, the c.763C>T and c.1030C>T variants predominantly produced aberrantly spliced transcripts, but in both cases, it disrupted the reading frame. Thus, despite the effect on splicing, they actually remain loss‐of‐function variants. Although we have not confirmed a ‘manufactured splice rescue’ mechanism for *PAX6* nonsense variants, its possible existence should be considered, especially when the severity of the phenotype does not correlate with a known causative variant.

Such detailed functional characterisation is critical for the accurate classification of variants, ultimately aiding in accurate genetic counselling, risk assessment and the development of potential therapeutic strategies. This approach is consistent with ongoing efforts to improve the granularity of genetic diagnosis by incorporating functional data into existing frameworks.

Our study validates the utility of minigene‐based assays in characterising splicing variants within the *PAX6* gene. However, it is important to acknowledge that this method has limitations, including those related to tissue‐specific differences in splicing patterns. Genome‐wide analysis has revealed that 10%–30% of alternatively spliced human genes exhibit tissue‐specific splice forms [63]. So, while HEK293T cells are commonly used for splicing analysis, they may not accurately reflect splicing events in the eye [64, 65]. On the other hand, Tarilonte et al. demonstrated concordance in the outcomes of minigene splicing analysis of *PAX6* variants conducted both in HEK‐293 and retinal ARPE‐19 cell lines. Furthermore, the results of the minigene splicing assay exhibited a concordance with the direct analysis of RNA from patient‐derived lymphocytes [49].

The results of the minigene splicing analysis can be used to refine the pathogenicity classification of the variants studied here by adding the ACMG PS3/BS3 criteria. However, multiple splicing events and variable residual expression of the wild‐type transcript may complicate pathogenicity assessment in some cases. To determine the level of residual wild‐type transcript expression that is insufficient to prevent disease development, we analysed the intronic variant c.357+5G>A. This variant has been reported in several unrelated families as a cause of congenital aniridia. Its pathogenicity is well established, with no proposed mechanism other than its effect on splicing. Our functional analysis revealed ~ 34% residual expression of the wild‐type transcript, suggesting that this level still leads to the disease onset. Based on these findings, we confidently applied the PS3 criterion to all variants with comparable or lower levels of residual wild‐type expression (Table 2). However, we exercised caution in our classification; therefore, the PS3 criterion was not applied to the remaining variants despite observed splicing effects, as they require further validation.

In conclusion, our findings contribute to a deeper understanding of *PAX6*‐related pathogenic mechanisms, offering a robust framework for future studies and enhancing the precision of genetic counselling and therapeutic development. By highlighting the prevalence and complexity of spliceogenic variants, we emphasise the importance of functional validation in genetic diagnostics.

## Author Contributions

**Kseniya Davydenko:** formal analysis (equal), investigation (equal), validation (equal), writing – original draft (equal). **Alexandra Filatova:** conceptualization (equal), data curation (equal), formal analysis (equal), methodology (equal), writing – review and editing (equal). **Mikhail Skoblov:** conceptualization (equal), data curation (equal), formal analysis (equal), supervision (equal), writing – review and editing (equal).

## Ethics Statement

The manuscript does not contain clinical studies or patient data.

## Conflicts of Interest

The authors declare no conflicts of interest.

## Supporting information


**FIGURE S1.** Development and functional validation of wild‐type minigene constructs for all coding exons of the *PAX6* gene. A. Design of a minigene plasmid. B. Electrophoresis of RT‐PCR products derived from HEK293 cells transfected with wild‐type minigene. C. Sanger sequencing of RT‐PCR product. D. Fragment analysis of RT‐PCR product by capillary electrophoresis. The identified transcript corresponds to a blue peak and their size is indicated by an asterisk.

**FIGURE S2.** Functional analysis of all selected PAX6 variants. A. The schematic diagram of the minigene construction and the localisation of intronic and exonic variants selected for minigene splicing assay. B. Electrophoresis of RT‐PCR products derived from HEK293 cells transfected with wild‐type and variant‐containing minigenes. C. Left to right: variant name following Human Genome Variation Society (HGVS) guidelines with reference NM_000280.4; Schematic representation of the splicing events observed by targeted next‐generation sequencing of RT‐PCR product; fragment analysis of RT‐PCR demonstrates the observed isoforms ratio.

**FIGURE S3.** Repetitions of the fragment analysis of RT‐PCR products from RNA isolated from HEK293T cells 48 h after transfection demonstrated the reproducibility of the obtained data. The first page shows examples with three replicates. Next, data with two replicates are provided for all studied variants.

**TABLE S1.** Primers used for cloning, mutagenesis and RT‐PCR analysis.

## Data Availability

All data generated or analysed during this study are included in this published article and its Supporting Information files.
