# Supplementary material for: Assessing Splicing Variants in the PAX6 Gene: A Comprehensive Minigene Approach
Source: J Cell Mol Med. 2025 Mar 25;29(6):e70459. doi: 10.1111/jcmm.70459 (PMC11936725; doi:10.1111/jcmm.70459)

Minigene construction of exon 4

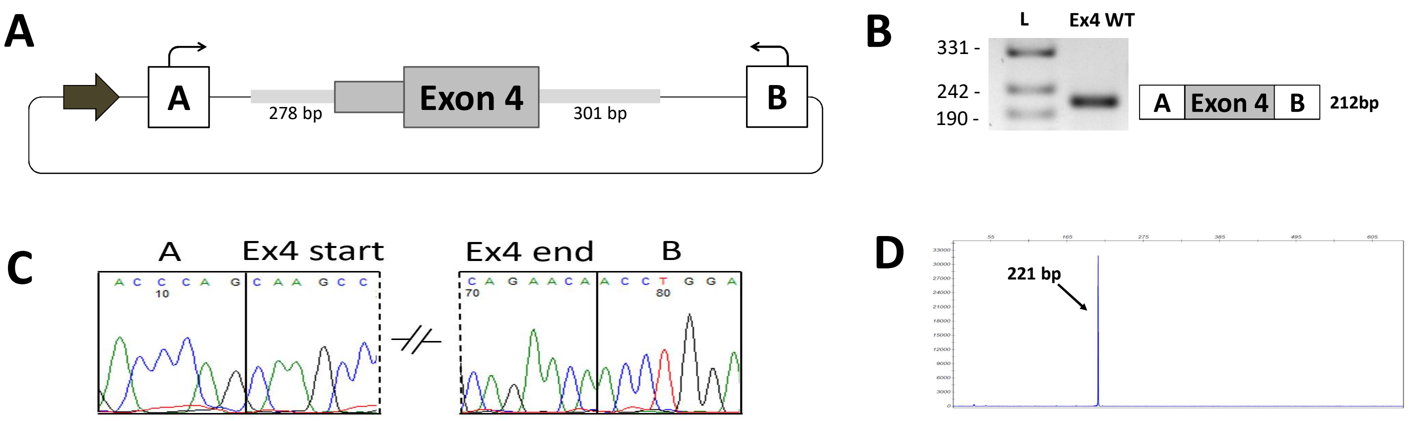

Minigene construction of exon 5

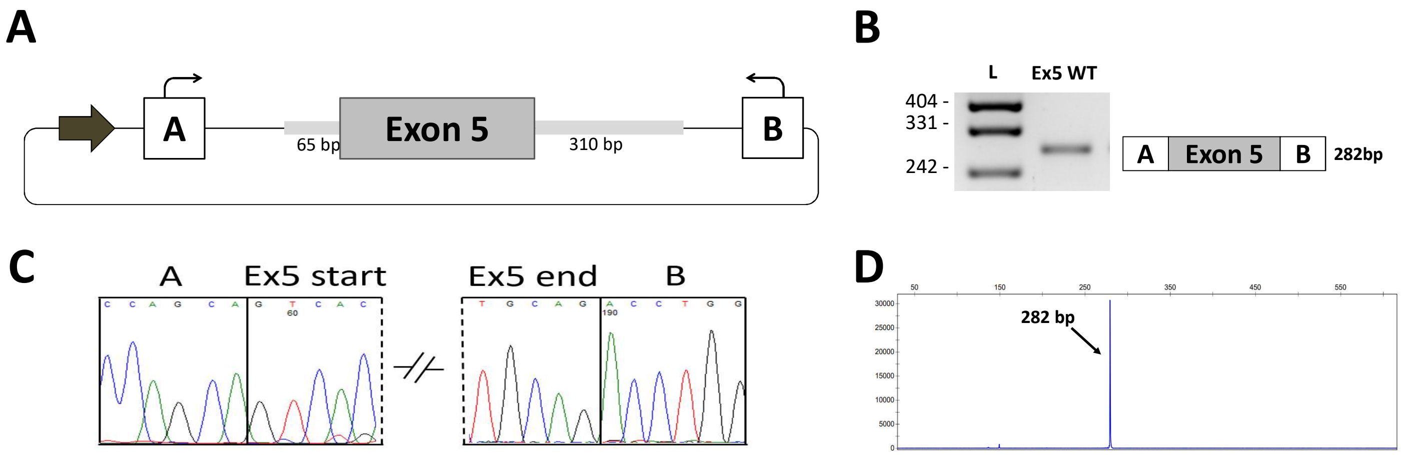

Minigene construction of exon 6

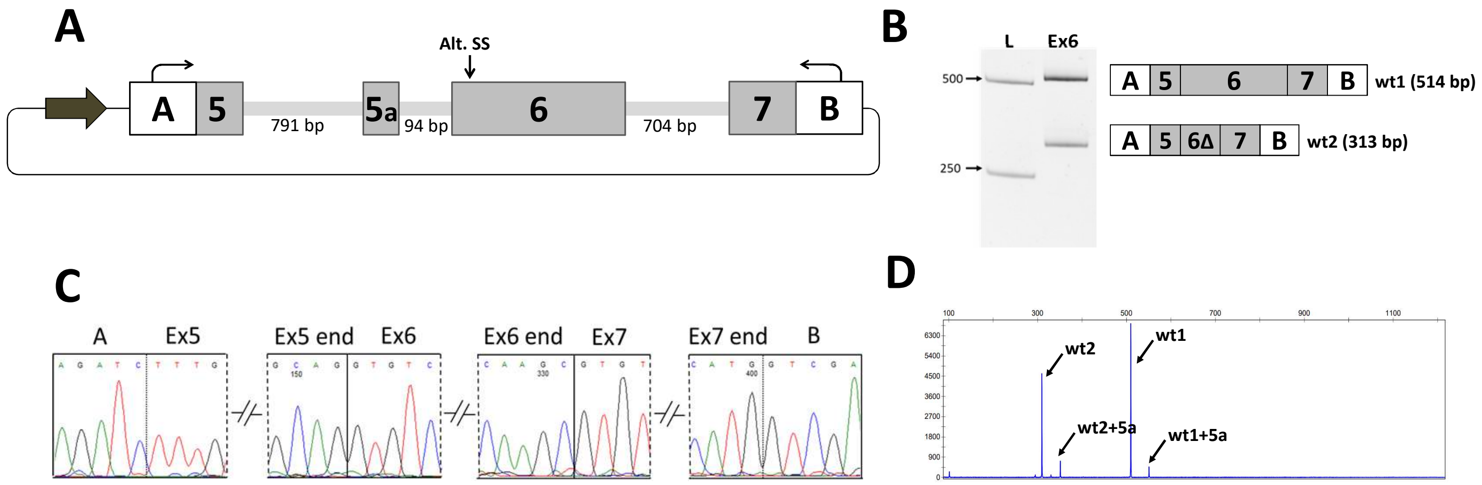

Minigene construction of exon 7

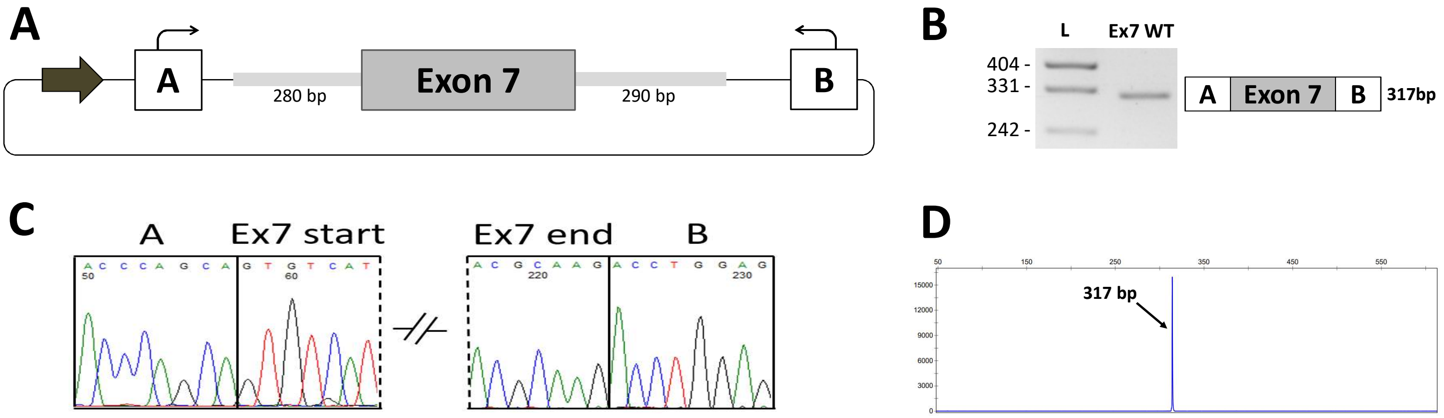

Minigene construction of exon 8

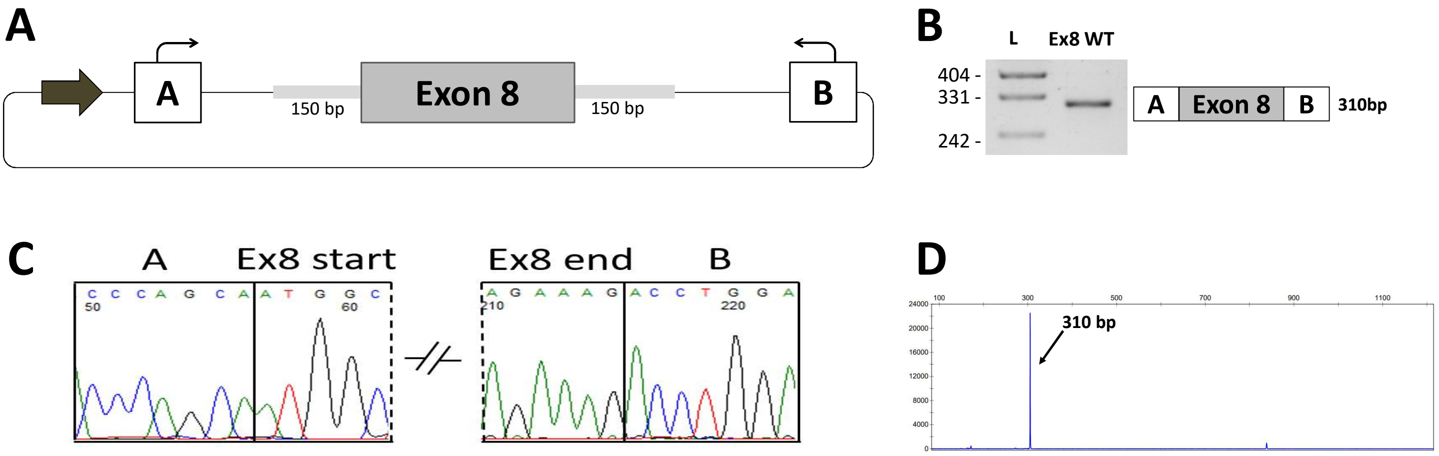

Minigene construction of exons 9-10-11

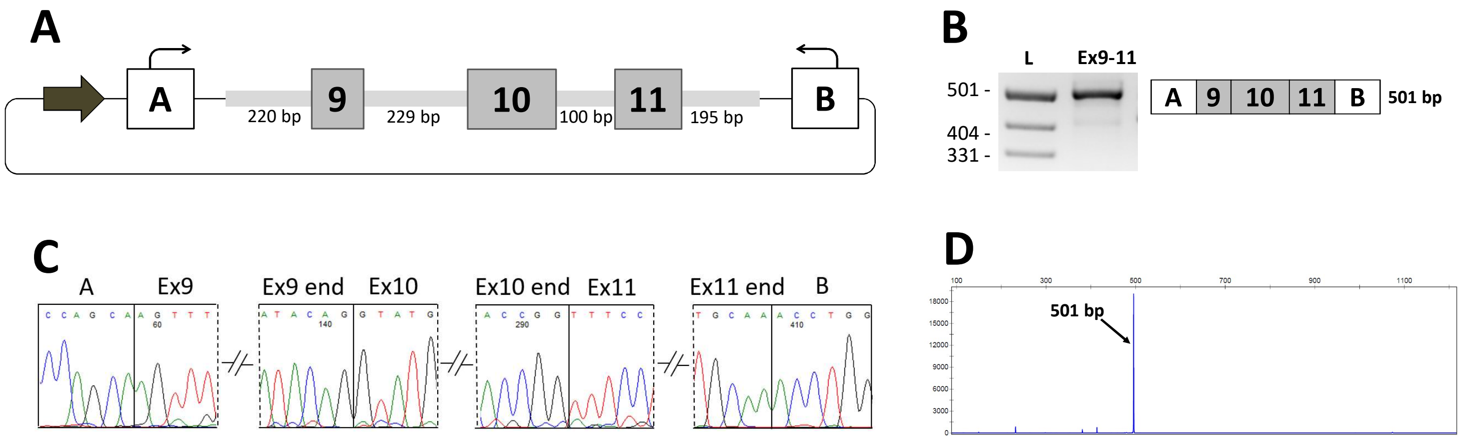

Minigene construction of exon 12

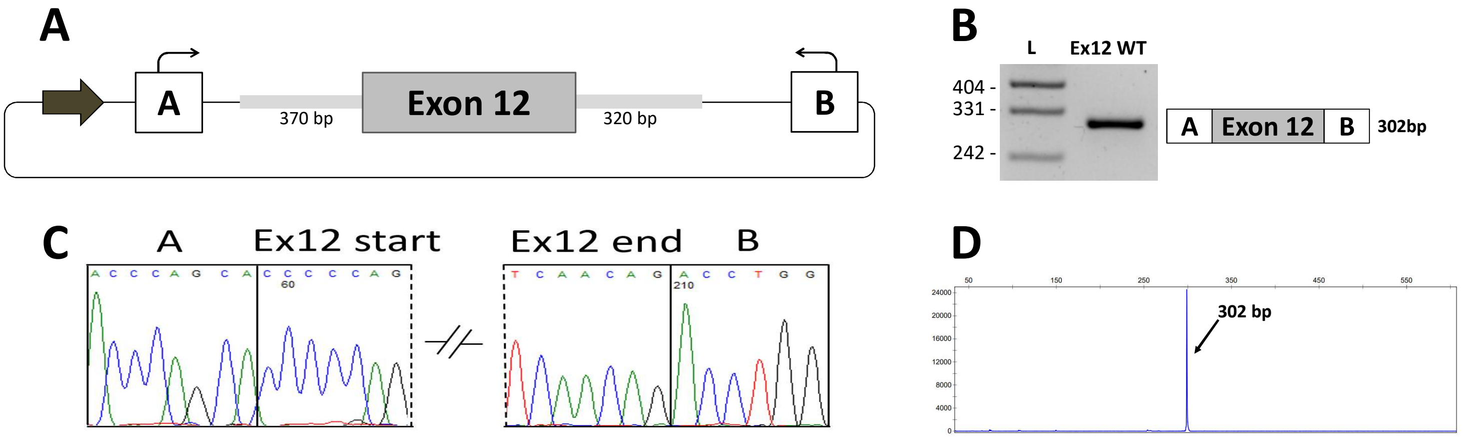

Minigene construction of exon 13

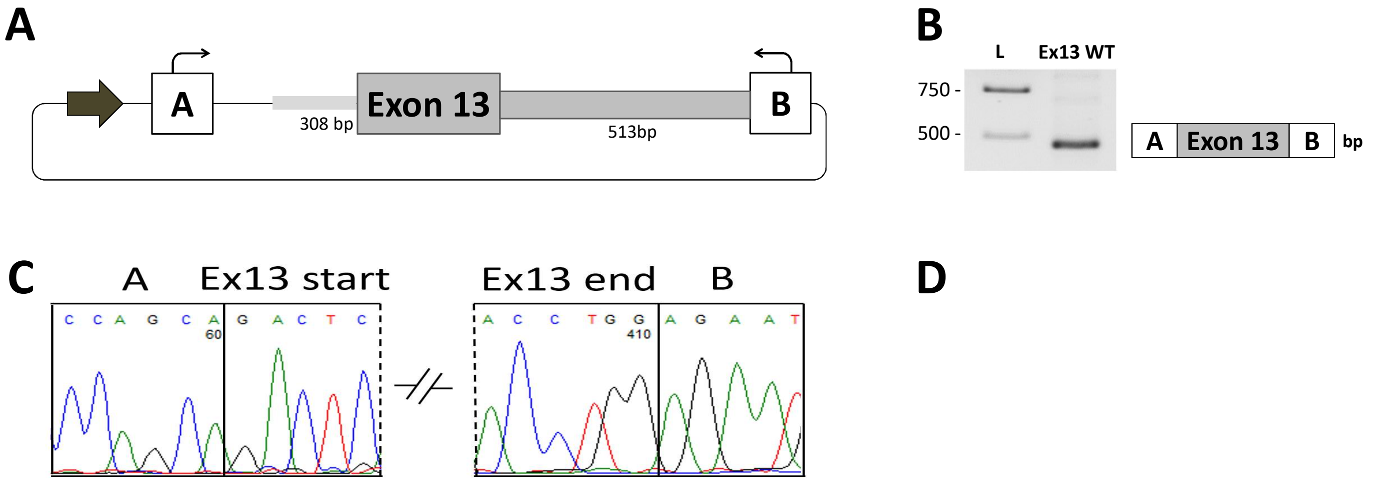

Supplement: Supplementary file 1 — FIGURE S1. Development and functional validation of wild‐type minigene constructs for all coding exons of the PAX6 gene. A. Design of a minigene plasmid. B. Electrophoresis of RT‐PCR products derived from HEK293 cells transfected with wild‐type minigene. C. Sanger sequencing of RT‐PCR product. D. Fragment analysis of RT‐PCR product by capillary electrophoresis. The identified transcript corresponds to a blue peak and their size is indicated by an asterisk. [file JCMM-29-e70459-s001.pdf]
