# Supplementary material for: Assessing Splicing Variants in the PAX6 Gene: A Comprehensive Minigene Approach
Source: J Cell Mol Med. 2025 Mar 25;29(6):e70459. doi: 10.1111/jcmm.70459 (PMC11936725; doi:10.1111/jcmm.70459)
Supplement: Supplementary file 2 — FIGURE S2. Functional analysis of all selected PAX6 variants. A. The schematic diagram of the minigene construction and the localisation of intronic and exonic variants selected for minigene splicing assay. B. Electrophoresis of RT‐PCR products derived from HEK293 cells transfected with wild‐type and variant‐containing minigenes. C. Left to right: variant name following Human Genome Variation Society (HGVS) guidelines with reference NM_000280.4; Schematic representation of the splicing events observed by targeted next‐generation sequencing of RT‐PCR product; fragment analysis of RT‐PCR demonstrates the observed isoforms ratio. [file JCMM-29-e70459-s004.pdf]

Exon 5

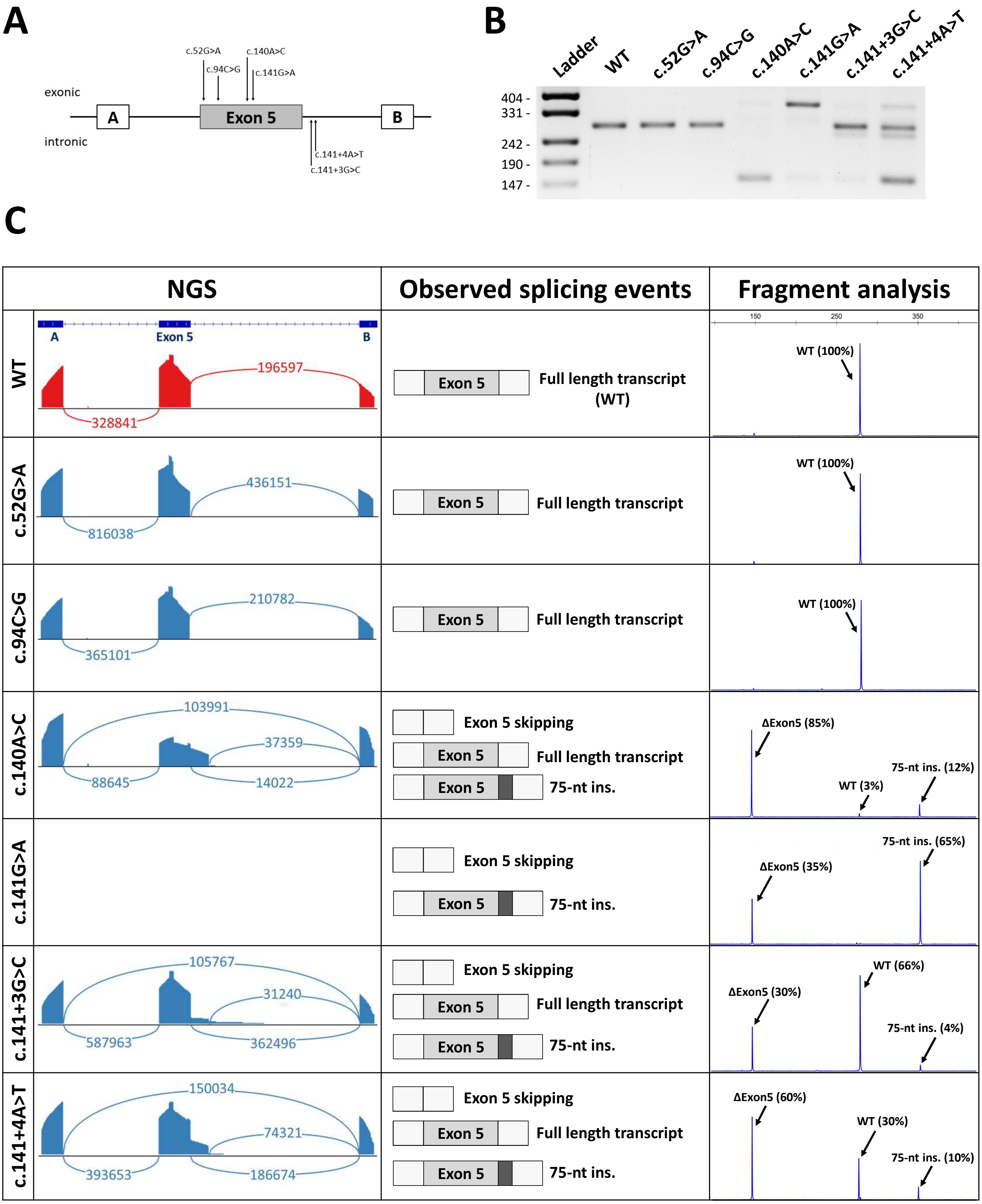
A
exonic
intronic
A
Exon 5
B
c.52G>A
c.94C>G
c.140A>C
c.141G>A
c.141+4A>T
c.141+3G>C
B
Ladder
WT
c.52G>A
c.94C>G
c.140A>C
c.141G>A
c.141+3G>C
c.141+4A>T
404 -
331 -
242 -
190 -
147 -
C
NGS
Observed splicing events
Fragment analysis
WT
A
Exon 5
B
196597
328841
Exon 5
Full length transcript (WT)
150
250
350
WT (100%)
c.52G>A
436151
816038
Exon 5
Full length transcript
WT (100%)
c.94C>G
210782
365101
Exon 5
Full length transcript
WT (100%)
c.140A>C
103991
37359
88645
14022
Exon 5 skipping
Exon 5
Full length transcript
Exon 5
75-nt ins.
ΔExon5 (85%)
WT (3%)
75-nt ins. (12%)
c.141G>A
Exon 5 skipping
Exon 5
75-nt ins.
ΔExon5 (35%)
75-nt ins. (65%)
c.141+3G>C
105767
31240
587963
362496
Exon 5 skipping
Exon 5
Full length transcript
Exon 5
75-nt ins.
ΔExon5 (30%)
WT (66%)
75-nt ins. (4%)
c.141+4A>T
150034
74321
393653
186674
Exon 5 skipping
Exon 5
Full length transcript
Exon 5
75-nt ins.
ΔExon5 (60%)
WT (30%)
75-nt ins. (10%)

Exon 6

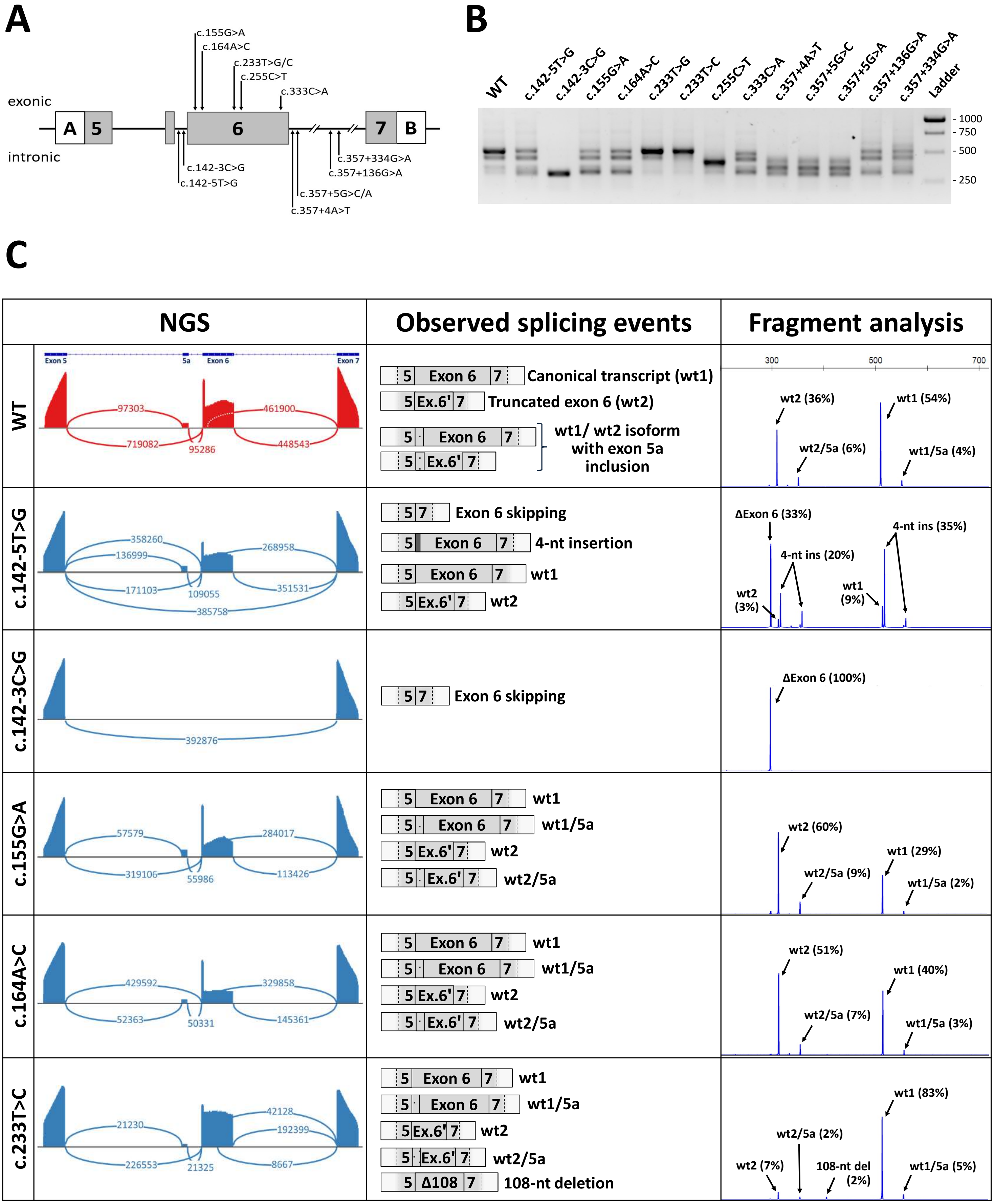
A
exonic
intronic
A
5
6
7
B
c.155G>A
c.164A>C
c.233T>G/C
c.255C>T
c.333C>A
c.142-3C>G
c.142-5T>G
c.357+334G>A
c.357+136G>A
c.357+5G>C/A
c.357+4A>T
B
WT
c.142-5T>G
c.142-3C>G
c.155G>A
c.164A>C
c.233T>G
c.233T>C
c.255C>T
c.333C>A
c.357+4A>T
c.357+5G>C
c.357+5G>A
c.357+136G>A
c.357+334G>A
Ladder
- 1000
- 750
- 500
- 250
C
NGS
Observed splicing events
Fragment analysis
WT
Exon 5
5a
Exon 6
Exon 7
97303
461900
719082
95286
448543
5 Exon 6 7 Canonical transcript (wt1)
5 Ex.6' 7 Truncated exon 6 (wt2)
5 Exon 6 7
5 Ex.6' 7
wt1/ wt2 isoform with exon 5a inclusion
300
500
700
wt2 (36%)
wt1 (54%)
wt2/5a (6%)
wt1/5a (4%)
c.142-5T>G
358260
136999
268958
171103
109055
351531
385758
5 7 Exon 6 skipping
5 Exon 6 7 4-nt insertion
5 Exon 6 7 wt1
5 Ex.6' 7 wt2
ΔExon 6 (33%)
4-nt ins (35%)
4-nt ins (20%)
wt2 (3%)
wt1 (9%)
c.142-3C>G
392876
5 7 Exon 6 skipping
ΔExon 6 (100%)
c.155G>A
57579
284017
319106
55986
113426
5 Exon 6 7 wt1
5 Exon 6 7 wt1/5a
5 Ex.6' 7 wt2
5 Ex.6' 7 wt2/5a
wt2 (60%)
wt1 (29%)
wt2/5a (9%)
wt1/5a (2%)
c.164A>C
429592
329858
52363
50331
145361
5 Exon 6 7 wt1
5 Exon 6 7 wt1/5a
5 Ex.6' 7 wt2
5 Ex.6' 7 wt2/5a
wt2 (51%)
wt1 (40%)
wt2/5a (7%)
wt1/5a (3%)
c.233T>C
21230
42128
192399
226553
21325
8667
5 Exon 6 7 wt1
5 Exon 6 7 wt1/5a
5 Ex.6' 7 wt2
5 Ex.6' 7 wt2/5a
5 Δ108 7 108-nt deletion
wt1 (83%)
wt2/5a (2%)
wt2 (7%)
108-nt del (2%)
wt1/5a (5%)

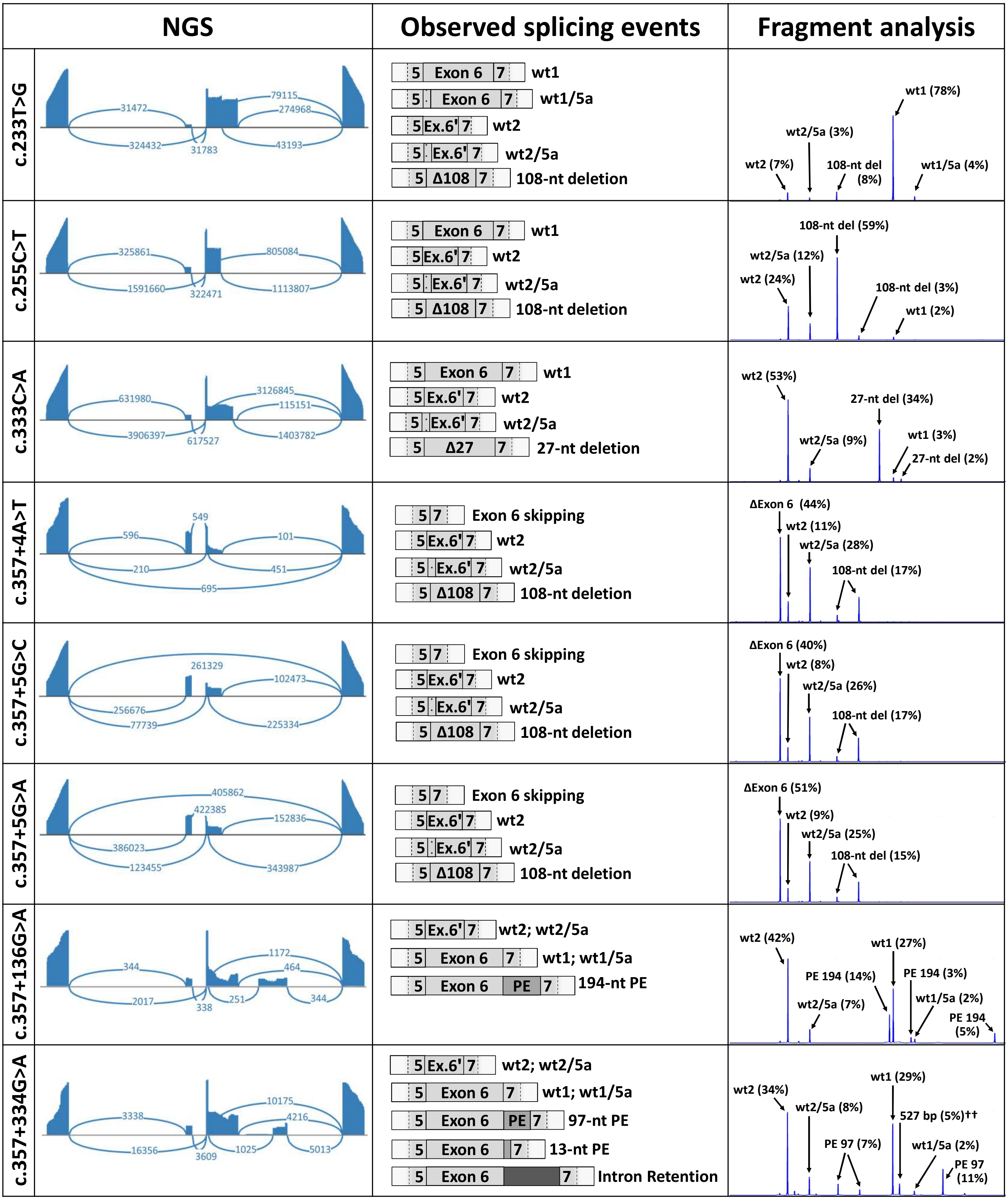
NGS
Observed splicing events
Fragment analysis
c.233T>G
31472
324432
31783
79115
274968
43193
5 Exon 6 7 wt1
5 Exon 6 7 wt1/5a
5 Ex.6' 7 wt2
5 Ex.6' 7 wt2/5a
5 Δ108 7 108-nt deletion
wt1 (78%)
wt2/5a (3%)
wt2 (7%)
108-nt del (8%)
wt1/5a (4%)
c.255C>T
325861
1591660
322471
805084
1113807
5 Exon 6 7 wt1
5 Ex.6' 7 wt2
5 Ex.6' 7 wt2/5a
5 Δ108 7 108-nt deletion
108-nt del (59%)
wt2/5a (12%)
wt2 (24%)
108-nt del (3%)
wt1 (2%)
c.333C>A
631980
3906397
617527
3126845
115151
1403782
5 Exon 6 7 wt1
5 Ex.6' 7 wt2
5 Ex.6' 7 wt2/5a
5 Δ27 7 27-nt deletion
wt2 (53%)
27-nt del (34%)
wt2/5a (9%)
wt1 (3%)
27-nt del (2%)
c.357+4A>T
596
549
101
210
451
695
5 7 Exon 6 skipping
5 Ex.6' 7 wt2
5 Ex.6' 7 wt2/5a
5 Δ108 7 108-nt deletion
ΔExon 6 (44%)
wt2 (11%)
wt2/5a (28%)
108-nt del (17%)
c.357+5G>C
261329
102473
256676
77739
225334
5 7 Exon 6 skipping
5 Ex.6' 7 wt2
5 Ex.6' 7 wt2/5a
5 Δ108 7 108-nt deletion
ΔExon 6 (40%)
wt2 (8%)
wt2/5a (26%)
108-nt del (17%)
c.357+5G>A
405862
422385
152836
386023
123455
343987
5 7 Exon 6 skipping
5 Ex.6' 7 wt2
5 Ex.6' 7 wt2/5a
5 Δ108 7 108-nt deletion
ΔExon 6 (51%)
wt2 (9%)
wt2/5a (25%)
108-nt del (15%)
c.357+136G>A
344
2017
338
1172
464
251
344
5 Ex.6' 7 wt2; wt2/5a
5 Exon 6 7 wt1; wt1/5a
5 Exon 6 PE 7 194-nt PE
wt2 (42%)
wt1 (27%)
PE 194 (14%)
PE 194 (3%)
wt2/5a (7%)
wt1/5a (2%)
PE 194 (5%)
c.357+334G>A
3338
16356
3609
10175
4216
1025
5013
5 Ex.6' 7 wt2; wt2/5a
5 Exon 6 7 wt1; wt1/5a
5 Exon 6 PE 7 97-nt PE
5 Exon 6 7 13-nt PE
5 Exon 6 7 Intron Retention
wt1 (29%)
wt2 (34%)
wt2/5a (8%)
527 bp (5%)††
PE 97 (7%)
wt1/5a (2%)
PE 97 (11%)

Exon 7

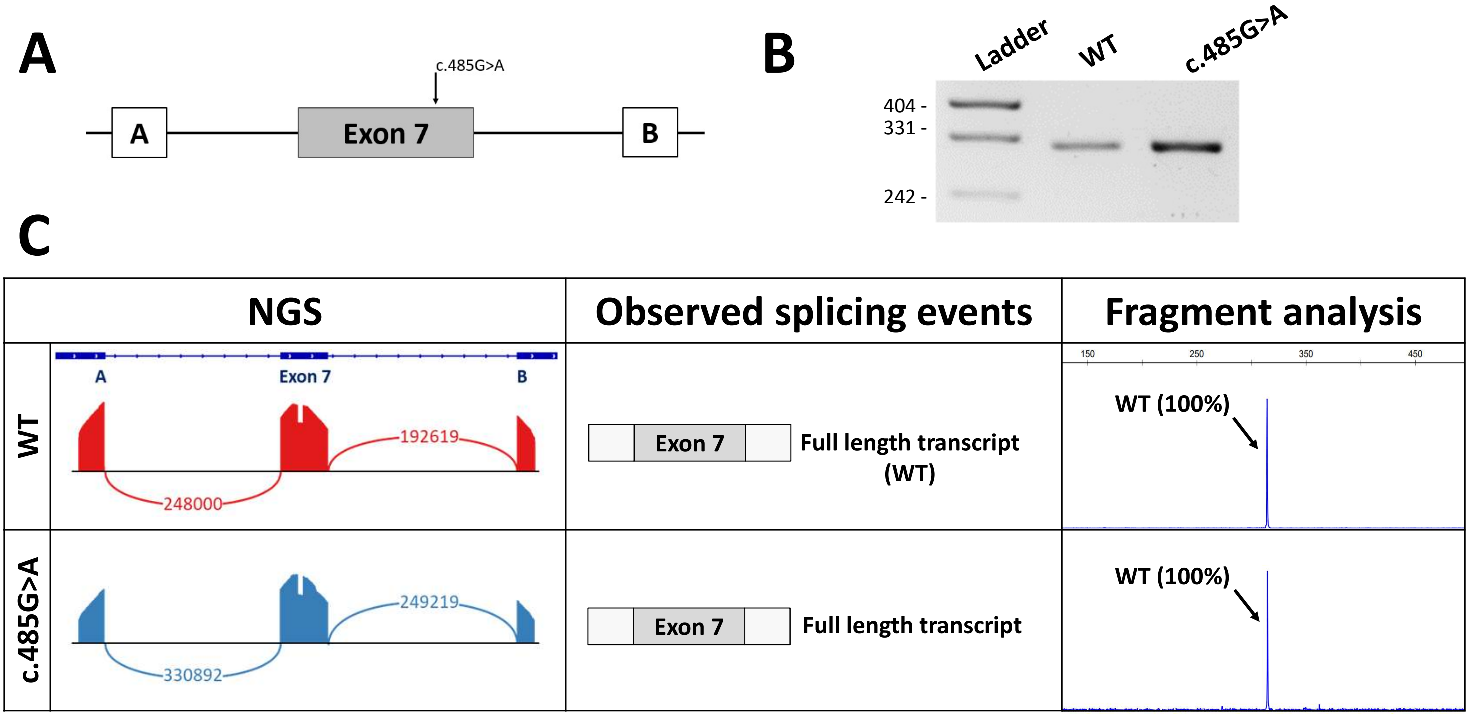
A
c.485G>A
A
Exon 7
B
B
Ladder
WT
c.485G>A
404 -
331 -
242 -
C
NGS
Observed splicing events
Fragment analysis
WT
A
Exon 7
B
192619
248000
Exon 7
Full length transcript
(WT)
150
250
350
450
WT (100%)
c.485G>A
249219
330892
Exon 7
Full length transcript
WT (100%)

Exon 8

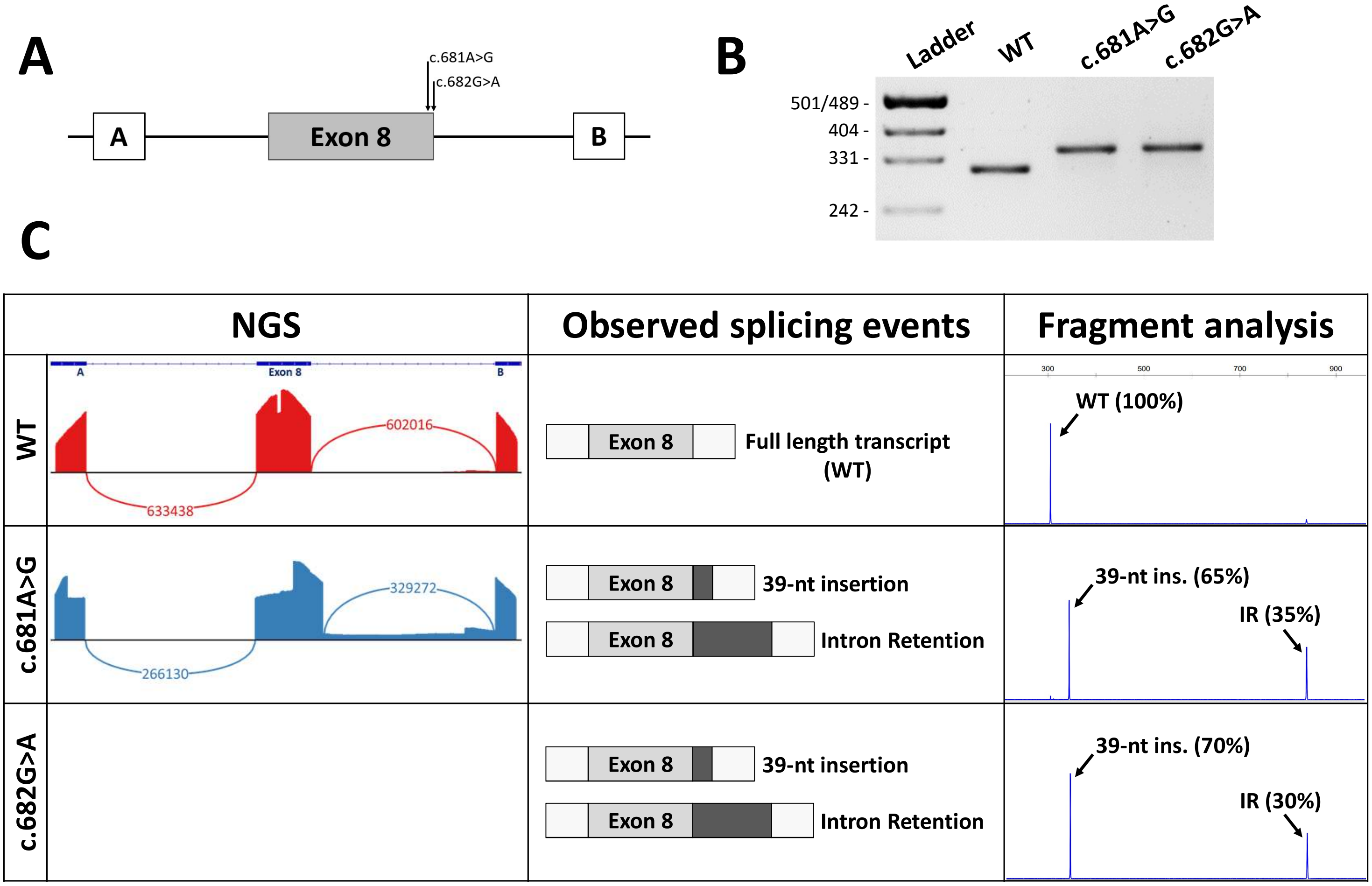
A
c.681A>G
c.682G>A
A
Exon 8
B
B
Ladder
WT
c.681A>G
c.682G>A
501/489 -
404 -
331 -
242 -
C
NGS
Observed splicing events
Fragment analysis
WT
A
Exon 8
B
602016
633438
Exon 8
Full length transcript
(WT)
300
500
700
900
WT (100%)
c.681A>G
329272
266130
Exon 8
39-nt insertion
Exon 8
Intron Retention
39-nt ins. (65%)
IR (35%)
c.682G>A
Exon 8
39-nt insertion
Exon 8
Intron Retention
39-nt ins. (70%)
IR (30%)

Exons 9–11

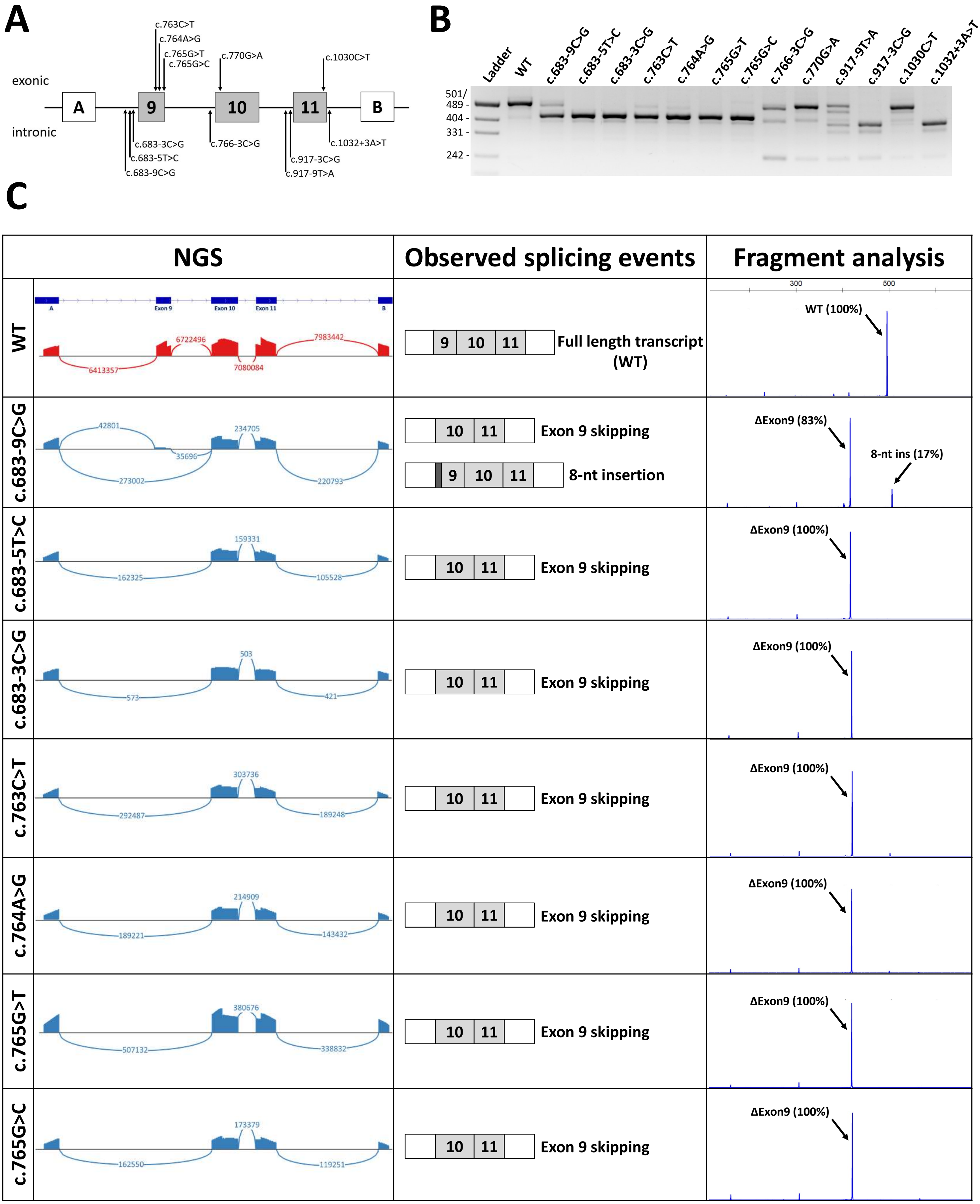
A
c.763C>T
c.764A>G
c.765G>T
c.765G>C
c.770G>A
c.1030C>T
exonic
A
9
10
11
B
intronic
c.683-3C>G
c.683-5T>C
c.683-9C>G
c.766-3C>G
c.1032+3A>T
c.917-3C>G
c.917-9T>A
B
Ladder
WT
c.683-9C>G
c.683-5T>C
c.683-3C>G
c.763C>T
c.764A>G
c.765G>T
c.765G>C
c.766-3C>G
c.770G>A
c.917-9T>A
c.917-3C>G
c.1030C>T
c.1032+3A>T
501/
489 -
404 -
331 -
242 -
C
NGS
Observed splicing events
Fragment analysis
WT
6722496
7983442
6413357
7080084
Full length transcript (WT)
WT (100%)
c.683-9C>G
42801
234705
35696
273002
220793
Exon 9 skipping
8-nt insertion
ΔExon9 (83%)
8-nt ins (17%)
c.683-5T>C
159331
162325
105528
Exon 9 skipping
ΔExon9 (100%)
c.683-3C>G
503
573
421
Exon 9 skipping
ΔExon9 (100%)
c.763C>T
303736
292487
189248
Exon 9 skipping
ΔExon9 (100%)
c.764A>G
214909
189221
143432
Exon 9 skipping
ΔExon9 (100%)
c.765G>T
380676
507132
338832
Exon 9 skipping
ΔExon9 (100%)
c.765G>C
173379
162550
119251
Exon 9 skipping
ΔExon9 (100%)

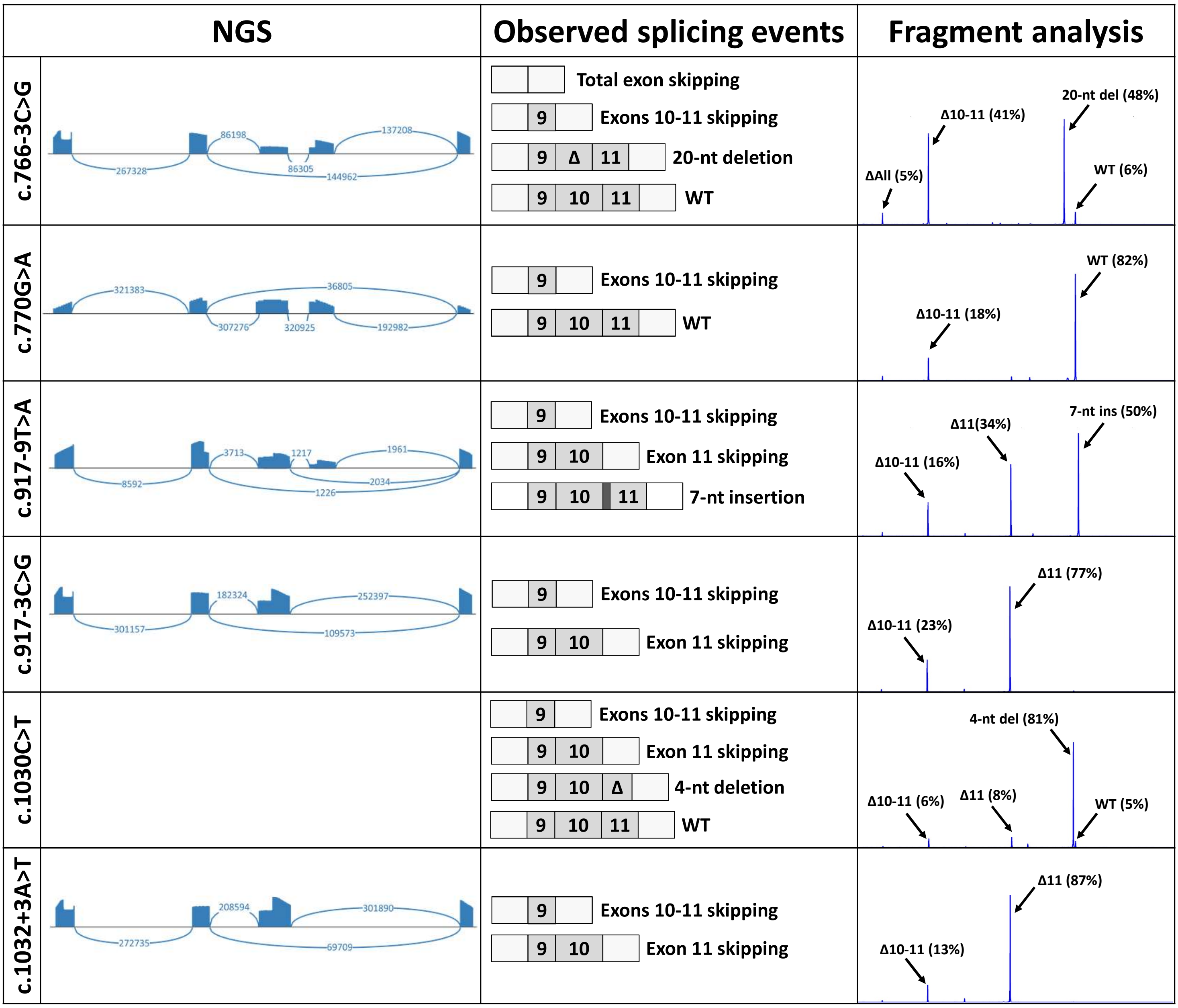
NGS
Observed splicing events
Fragment analysis
c.766-3C>G
86198
137208
267328
86305
144962
Total exon skipping
9
Exons 10-11 skipping
9
Δ
11
20-nt deletion
9
10
11
WT
20-nt del (48%)
Δ10-11 (41%)
ΔAll (5%)
WT (6%)
c.770G>A
321383
36805
307276
320925
192982
9
Exons 10-11 skipping
9
10
11
WT
WT (82%)
Δ10-11 (18%)
c.917-9T>A
3713
1217
1961
8592
2034
1226
9
Exons 10-11 skipping
9
10
Exon 11 skipping
9
10
11
7-nt insertion
7-nt ins (50%)
Δ11(34%)
Δ10-11 (16%)
c.917-3C>G
182324
252397
301157
109573
9
Exons 10-11 skipping
9
10
Exon 11 skipping
Δ11 (77%)
Δ10-11 (23%)
c.1030C>T
9
Exons 10-11 skipping
9
10
Exon 11 skipping
9
10
Δ
4-nt deletion
9
10
11
WT
4-nt del (81%)
Δ10-11 (6%)
Δ11 (8%)
WT (5%)
c.1032+3A>T
208594
301890
272735
69709
9
Exons 10-11 skipping
9
10
Exon 11 skipping
Δ11 (87%)
Δ10-11 (13%)

Exon 12

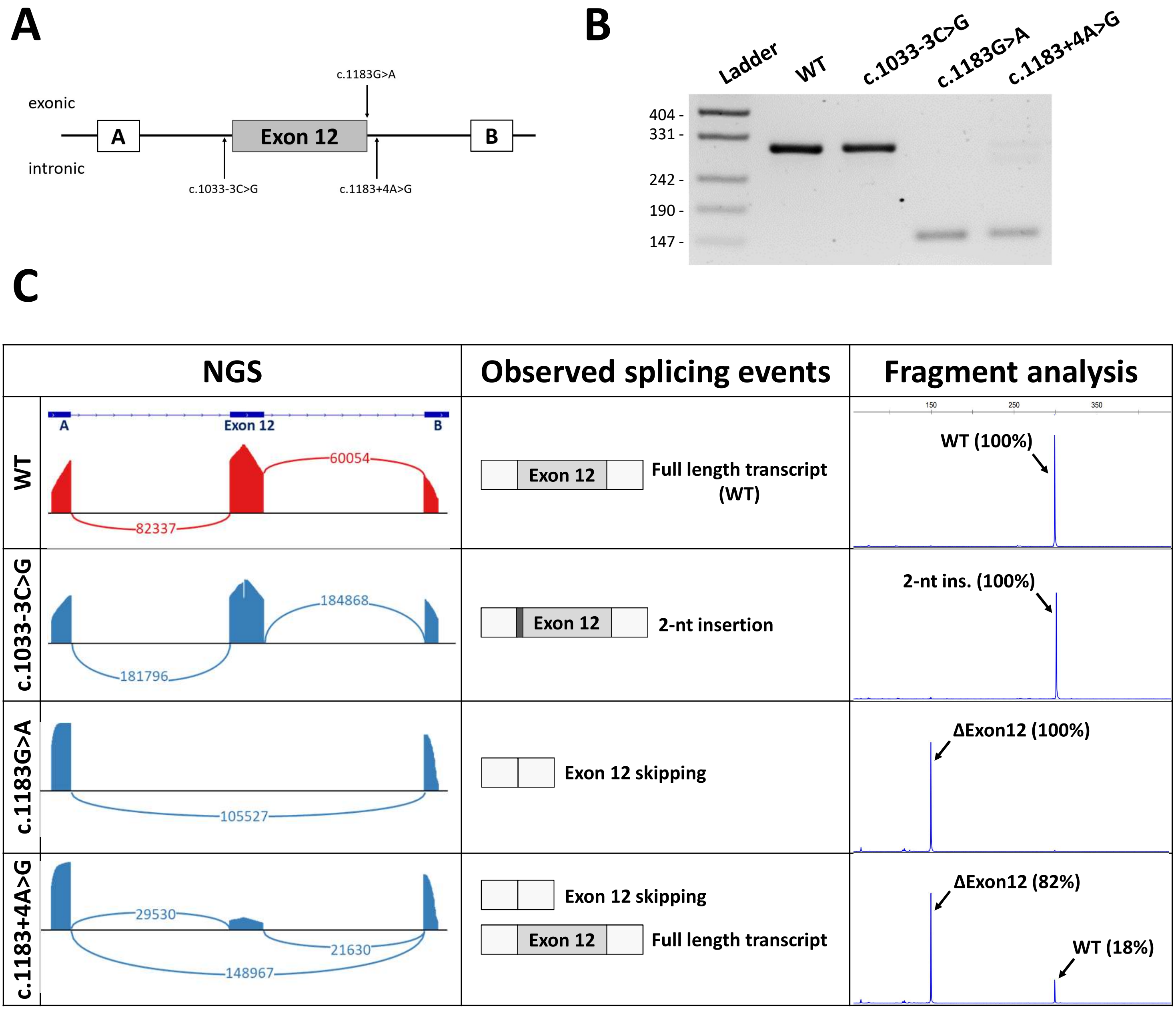
A
c.1183G>A
exonic
A
Exon 12
B
intronic
c.1033-3C>G
c.1183+4A>G
B
Ladder
WT
c.1033-3C>G
c.1183G>A
c.1183+4A>G
404 -
331 -
242 -
190 -
147 -
C
NGS
Observed splicing events
Fragment analysis
WT
A
Exon 12
B
60054
82337
Exon 12
Full length transcript (WT)
150
250
350
WT (100%)
c.1033-3C>G
184868
181796
Exon 12
2-nt insertion
2-nt ins. (100%)
c.1183G>A
105527
Exon 12 skipping
ΔExon12 (100%)
c.1183+4A>G
29530
21630
148967
Exon 12 skipping
Exon 12
Full length transcript
ΔExon12 (82%)
WT (18%)
