# Supplementary material for: Assessing Splicing Variants in the PAX6 Gene: A Comprehensive Minigene Approach
Source: J Cell Mol Med. 2025 Mar 25;29(6):e70459. doi: 10.1111/jcmm.70459 (PMC11936725; doi:10.1111/jcmm.70459)
Supplement: Supplementary file 3 — FIGURE S3. Repetitions of the fragment analysis of RT‐PCR products from RNA isolated from HEK293T cells 48 h after transfection demonstrated the reproducibility of the obtained data. The first page shows examples with three replicates. Next, data with two replicates are provided for all studied variants. [file JCMM-29-e70459-s003.pdf]

Exon 6

WT

FA Repetition-1	FA Repetition-2	FA Repetition-3

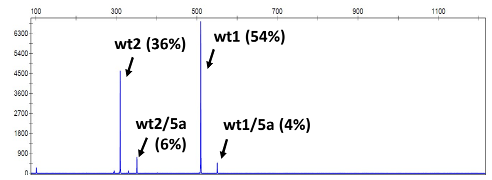

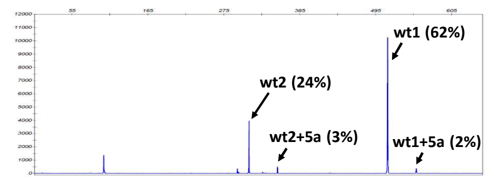

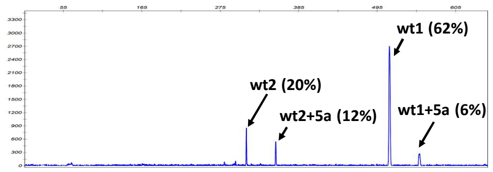

Exon 9-11

c.917-3C>G

FA Repetition-1	FA Repetition-2	FA Repetition-3

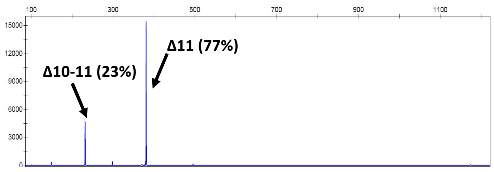

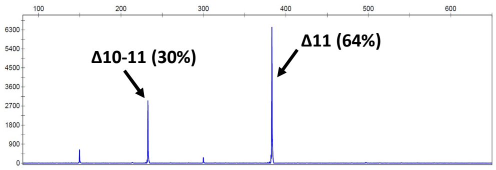

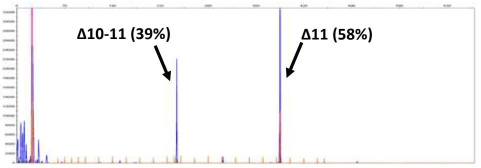

Exon 12

c.1183+4A>G

FA Repetition-1	FA Repetition-2	FA Repetition-3

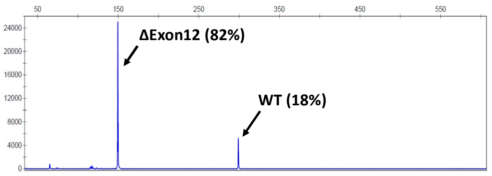

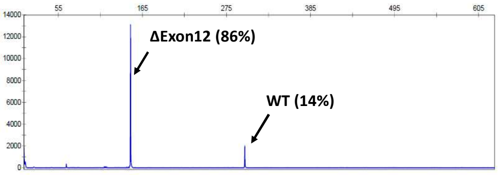

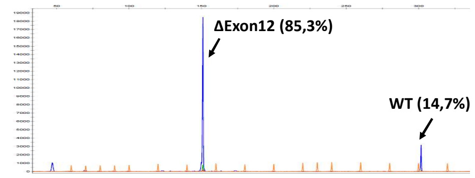

Exon 5

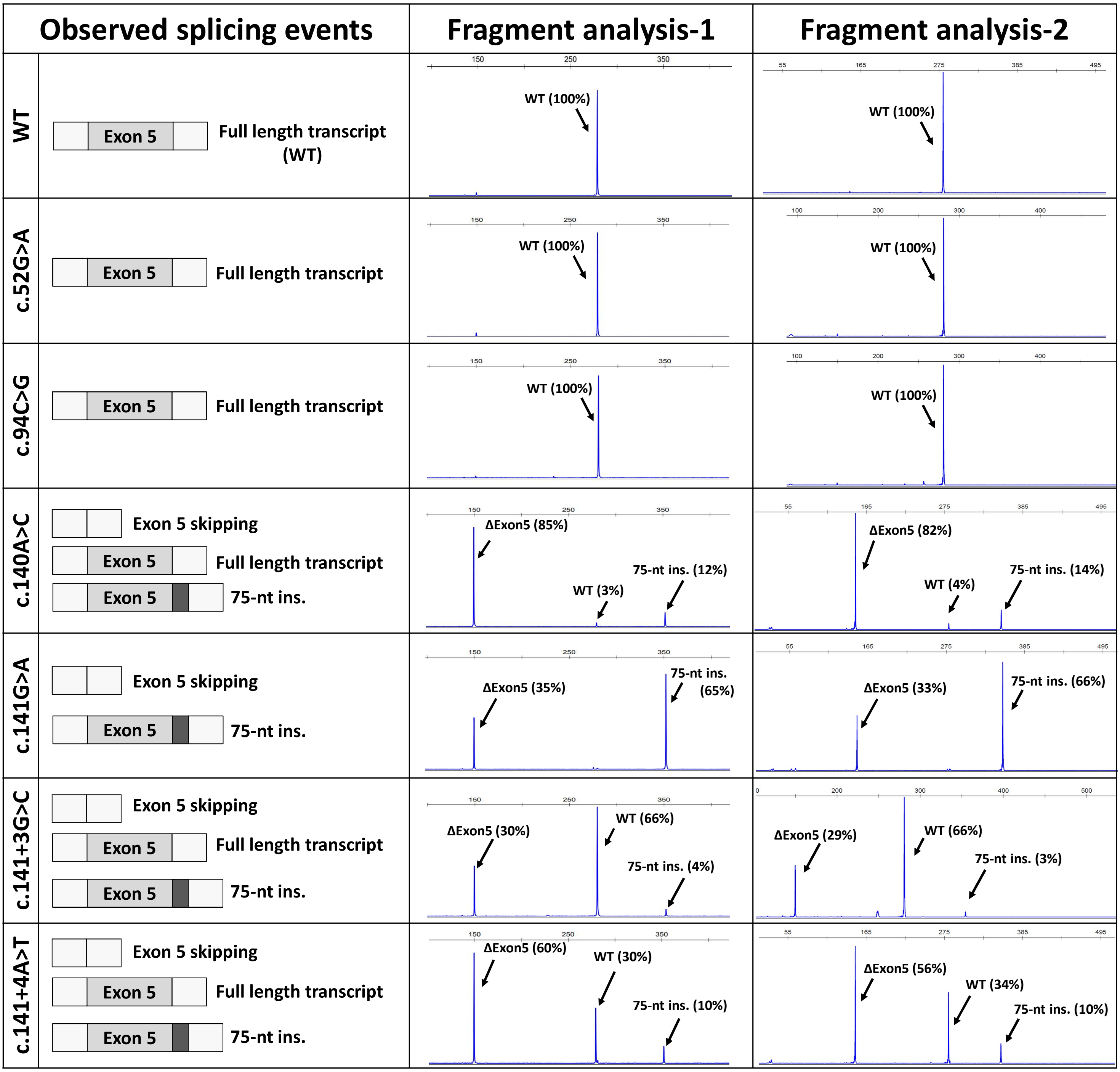

Observed splicing events
Fragment analysis-1
Fragment analysis-2
WT
Exon 5
Full length transcript (WT)
WT (100%)
c.52G>A
Exon 5
Full length transcript
WT (100%)
c.94C>G
Exon 5
Full length transcript
WT (100%)
c.140A>C
Exon 5 skipping
Exon 5
Full length transcript
Exon 5
75-nt ins.
ΔExon5 (85%)
WT (3%)
75-nt ins. (12%)
ΔExon5 (82%)
WT (4%)
75-nt ins. (14%)
c.141G>A
Exon 5 skipping
Exon 5
75-nt ins.
ΔExon5 (35%)
75-nt ins. (65%)
ΔExon5 (33%)
75-nt ins. (66%)
c.141+3G>C
Exon 5 skipping
Exon 5
Full length transcript
Exon 5
75-nt ins.
ΔExon5 (30%)
WT (66%)
75-nt ins. (4%)
ΔExon5 (29%)
WT (66%)
75-nt ins. (3%)
c.141+4A>T
Exon 5 skipping
Exon 5
Full length transcript
Exon 5
75-nt ins.
ΔExon5 (60%)
WT (30%)
75-nt ins. (10%)
ΔExon5 (56%)
WT (34%)
75-nt ins. (10%)

Exon 6

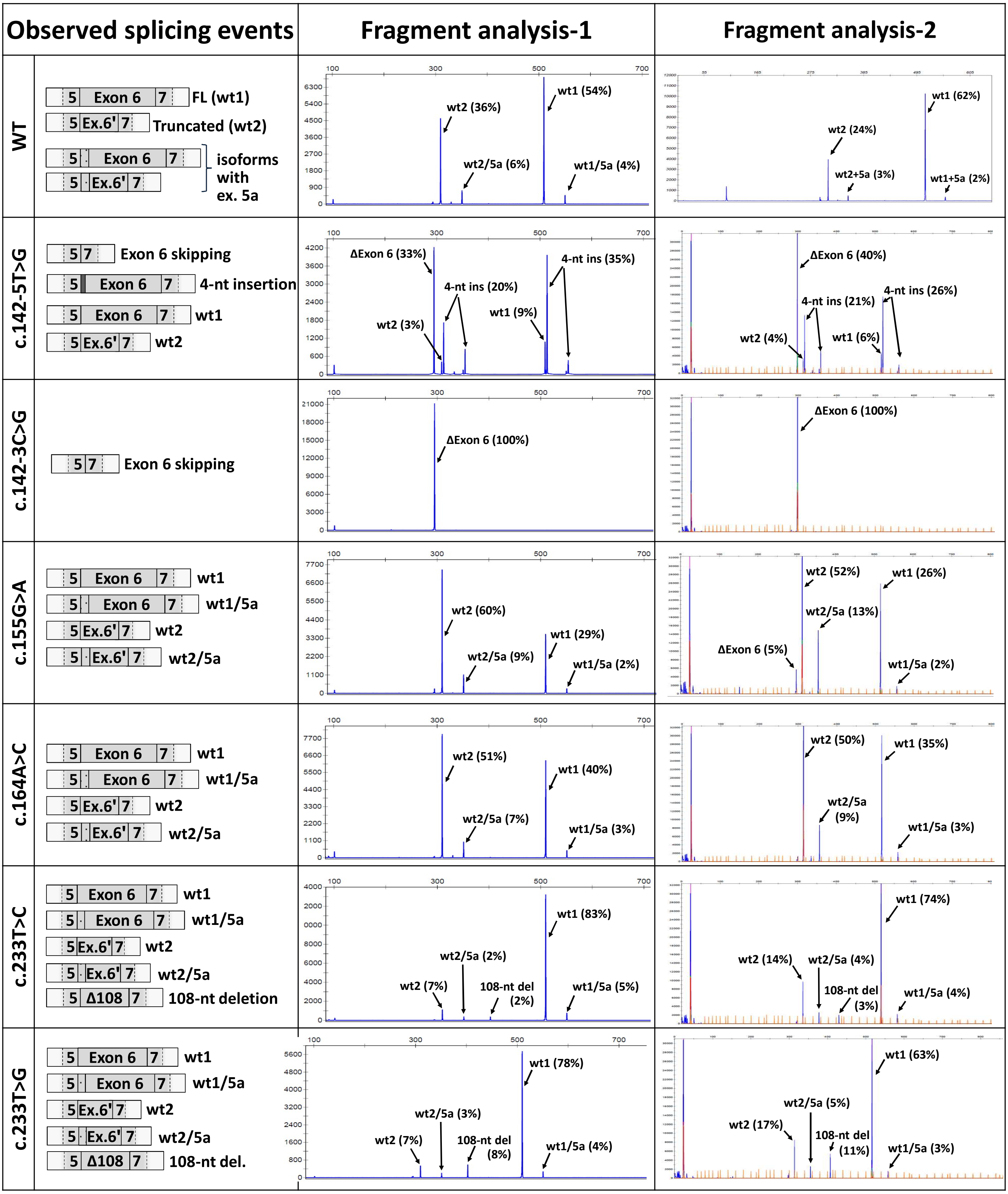

Observed splicing events
Fragment analysis-1
Fragment analysis-2
WT
5 Exon 6 7 FL (wt1)
5 Ex.6' 7 Truncated (wt2)
5 Exon 6 7
5 Ex.6' 7
isoforms with ex. 5a
wt1 (54%)
wt2 (36%)
wt2/5a (6%)
wt1/5a (4%)
wt1 (62%)
wt2 (24%)
wt2+5a (3%)
wt1+5a (2%)
c.142-5T>G
5 7 Exon 6 skipping
5 Exon 6 7 4-nt insertion
5 Exon 6 7 wt1
5 Ex.6' 7 wt2
ΔExon 6 (33%)
4-nt ins (35%)
4-nt ins (20%)
wt1 (9%)
wt2 (3%)
ΔExon 6 (40%)
4-nt ins (26%)
4-nt ins (21%)
wt2 (4%)
wt1 (6%)
c.142-3C>G
5 7 Exon 6 skipping
ΔExon 6 (100%)
ΔExon 6 (100%)
c.155G>A
5 Exon 6 7 wt1
5 Exon 6 7 wt1/5a
5 Ex.6' 7 wt2
5 Ex.6' 7 wt2/5a
wt2 (60%)
wt1 (29%)
wt2/5a (9%)
wt1/5a (2%)
wt2 (52%)
wt1 (26%)
wt2/5a (13%)
ΔExon 6 (5%)
wt1/5a (2%)
c.164A>C
5 Exon 6 7 wt1
5 Exon 6 7 wt1/5a
5 Ex.6' 7 wt2
5 Ex.6' 7 wt2/5a
wt2 (51%)
wt1 (40%)
wt2/5a (7%)
wt1/5a (3%)
wt2 (50%)
wt1 (35%)
wt2/5a (9%)
wt1/5a (3%)
c.233T>C
5 Exon 6 7 wt1
5 Exon 6 7 wt1/5a
5 Ex.6' 7 wt2
5 Ex.6' 7 wt2/5a
5 Δ108 7 108-nt deletion
wt1 (83%)
wt2/5a (2%)
wt2 (7%)
108-nt del (2%)
wt1/5a (5%)
wt1 (74%)
wt2 (14%)
wt2/5a (4%)
108-nt del (3%)
wt1/5a (4%)
c.233T>G
5 Exon 6 7 wt1
5 Exon 6 7 wt1/5a
5 Ex.6' 7 wt2
5 Ex.6' 7 wt2/5a
5 Δ108 7 108-nt del.
wt1 (78%)
wt2/5a (3%)
wt2 (7%)
108-nt del (8%)
wt1/5a (4%)
wt1 (63%)
wt2/5a (5%)
wt2 (17%)
108-nt del (11%)
wt1/5a (3%)

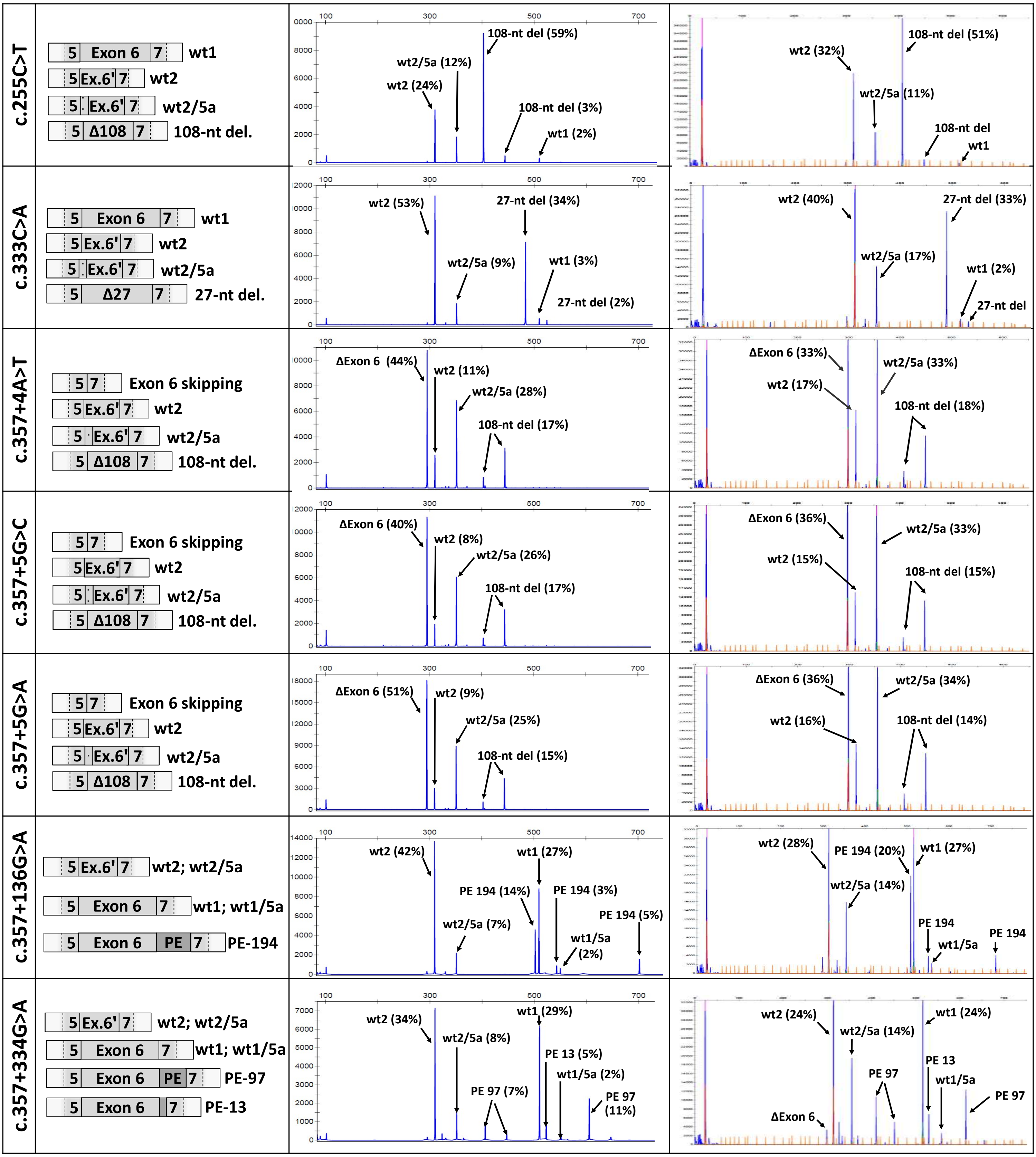

c.255C>T
5 Exon 6 7 wt1
5 Ex.6' 7 wt2
5 Ex.6' 7 wt2/5a
5 Δ108 7 108-nt del.
108-nt del (59%)
wt2/5a (12%)
wt2 (24%)
108-nt del (3%)
wt1 (2%)
wt2 (32%)
108-nt del (51%)
wt2/5a (11%)
108-nt del
wt1
c.333C>A
5 Exon 6 7 wt1
5 Ex.6' 7 wt2
5 Ex.6' 7 wt2/5a
5 Δ27 7 27-nt del.
wt2 (53%)
27-nt del (34%)
wt2/5a (9%)
wt1 (3%)
27-nt del (2%)
wt2 (40%)
27-nt del (33%)
wt2/5a (17%)
wt1 (2%)
27-nt del
c.357+4A>T
5 7 Exon 6 skipping
5 Ex.6' 7 wt2
5 Ex.6' 7 wt2/5a
5 Δ108 7 108-nt del.
ΔExon 6 (44%)
wt2 (11%)
wt2/5a (28%)
108-nt del (17%)
ΔExon 6 (33%)
wt2/5a (33%)
wt2 (17%)
108-nt del (18%)
c.357+5G>C
5 7 Exon 6 skipping
5 Ex.6' 7 wt2
5 Ex.6' 7 wt2/5a
5 Δ108 7 108-nt del.
ΔExon 6 (40%)
wt2 (8%)
wt2/5a (26%)
108-nt del (17%)
ΔExon 6 (36%)
wt2/5a (33%)
wt2 (15%)
108-nt del (15%)
c.357+5G>A
5 7 Exon 6 skipping
5 Ex.6' 7 wt2
5 Ex.6' 7 wt2/5a
5 Δ108 7 108-nt del.
ΔExon 6 (51%)
wt2 (9%)
wt2/5a (25%)
108-nt del (15%)
ΔExon 6 (36%)
wt2/5a (34%)
wt2 (16%)
108-nt del (14%)
c.357+136G>A
5 Ex.6' 7 wt2; wt2/5a
5 Exon 6 7 wt1; wt1/5a
5 Exon 6 PE 7 PE-194
wt2 (42%)
wt1 (27%)
PE 194 (14%)
PE 194 (3%)
PE 194 (5%)
wt2/5a (7%)
wt1/5a (2%)
wt2 (28%)
PE 194 (20%)
wt1 (27%)
wt2/5a (14%)
PE 194
wt1/5a
PE 194
c.357+334G>A
5 Ex.6' 7 wt2; wt2/5a
5 Exon 6 7 wt1; wt1/5a
5 Exon 6 PE 7 PE-97
5 Exon 6 7 PE-13
wt2 (34%)
wt1 (29%)
wt2/5a (8%)
PE 13 (5%)
wt1/5a (2%)
PE 97 (7%)
PE 97 (11%)
wt2 (24%)
wt1 (24%)
wt2/5a (14%)
PE 13
PE 97
wt1/5a
PE 97
ΔExon 6

Exon 7

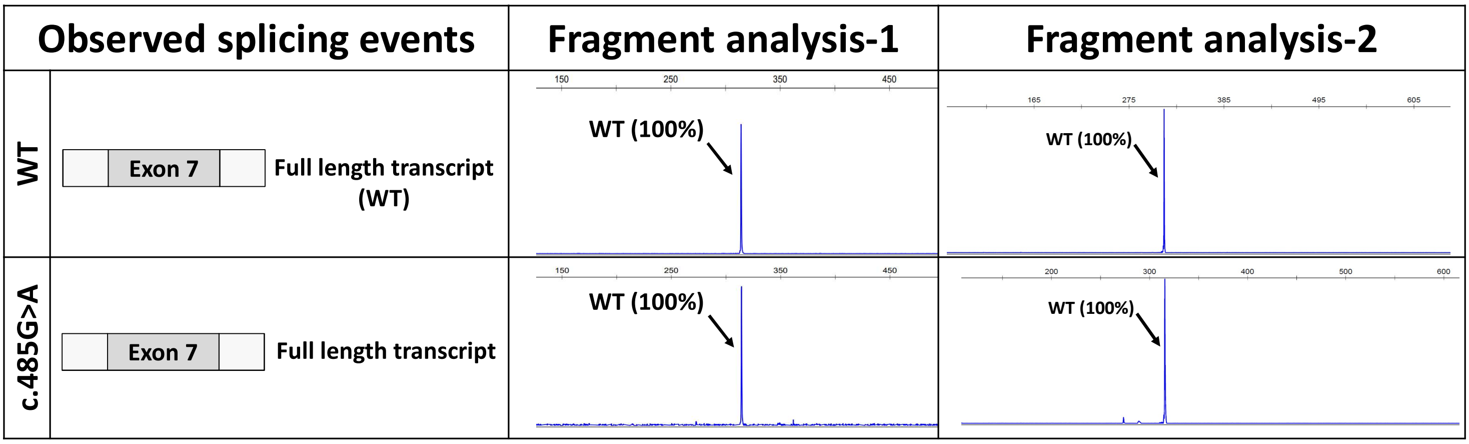

Observed splicing events
Fragment analysis-1
Fragment analysis-2
WT
Exon 7
Full length transcript (WT)
WT (100%)
WT (100%)
c.485G>A
Exon 7
Full length transcript
WT (100%)
WT (100%)

Exon 8

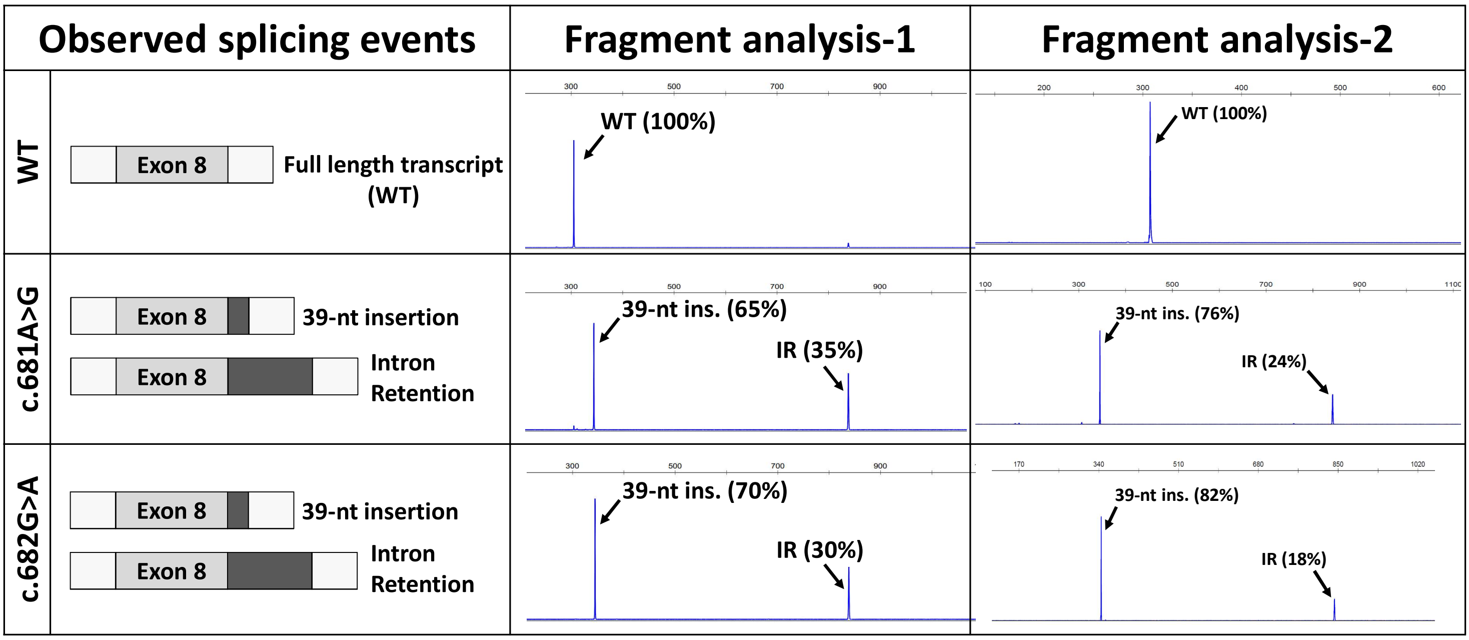

Observed splicing events
Fragment analysis-1
Fragment analysis-2
WT
Exon 8
Full length transcript (WT)
WT (100%)
WT (100%)
c.681A>G
Exon 8
39-nt insertion
Exon 8
Intron Retention
39-nt ins. (65%)
IR (35%)
39-nt ins. (76%)
IR (24%)
c.682G>A
Exon 8
39-nt insertion
Exon 8
Intron Retention
39-nt ins. (70%)
IR (30%)
39-nt ins. (82%)
IR (18%)

Exons 9–11

	Observed splicing events	Fragment analysis-1	Fragment analysis-2
WT	[| 9 | 10 | 11 |] Full length transcript (WT)	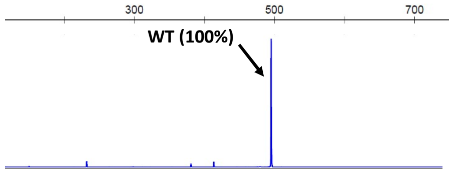	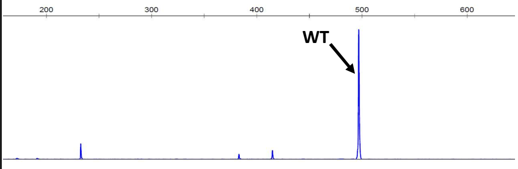
c.683-9C>G	[| 10 | 11 |] Exon 9 skipping<br>[| 9 | 10 | 11 |] 8-nt insertion	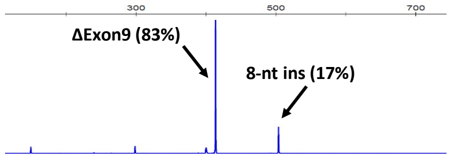	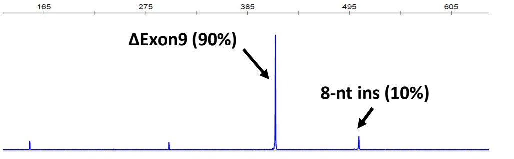
c.683-5T>C	[| 10 | 11 |] Exon 9 skipping	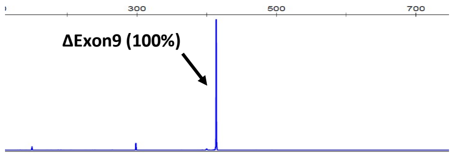	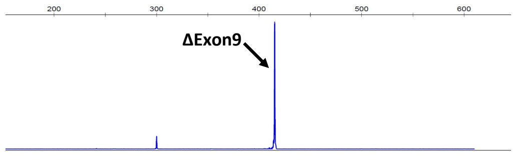
c.683-3C>G	[| 10 | 11 |] Exon 9 skipping	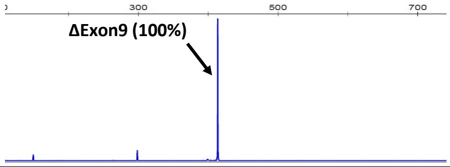	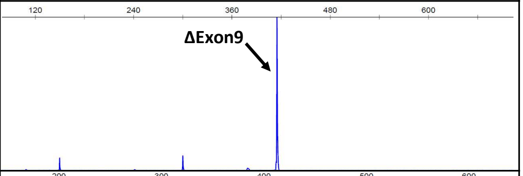
c.763C>T	[| 10 | 11 |] Exon 9 skipping	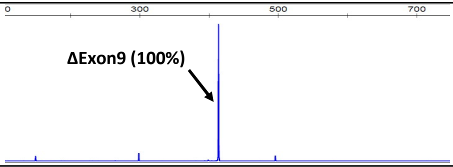	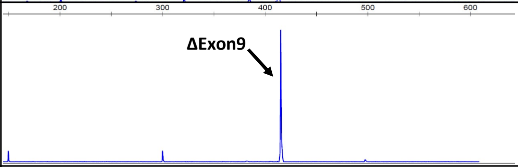
c.764A>G	[| 10 | 11 |] Exon 9 skipping	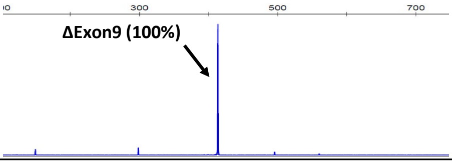	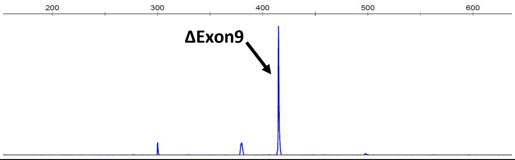
c.765G>T	[| 10 | 11 |] Exon 9 skipping	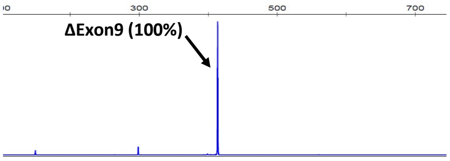	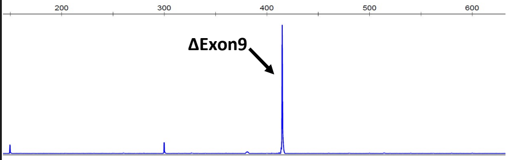
c.765G>C	[| 10 | 11 |] Exon 9 skipping	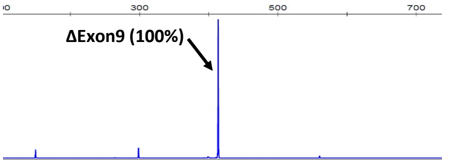	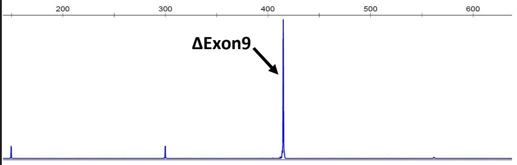
c.766-3C>G	[|] Total exon skipping<br>[| 9 |] Exons 10-11 skipping<br>[| 9 | Δ | 11 |] 20-nt deletion<br>[| 9 | 10 | 11 |] WT	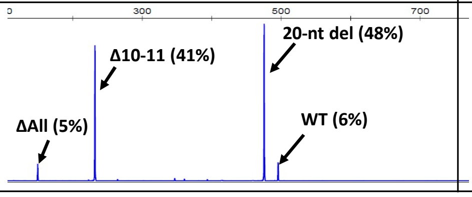	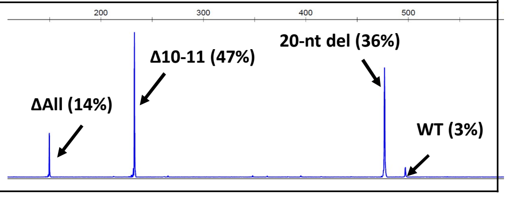

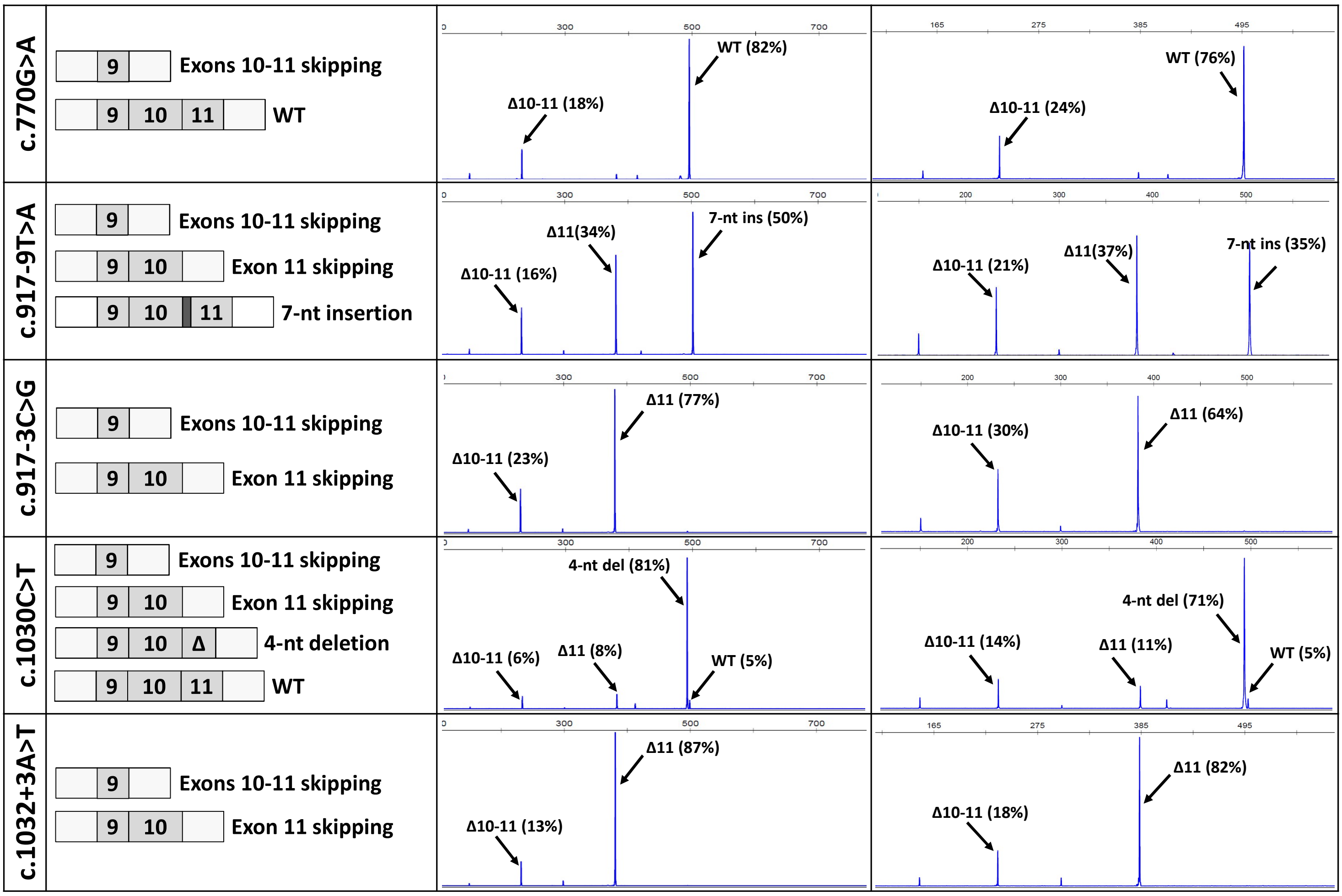

c.770G>A
9
Exons 10-11 skipping
9 10 11
WT
Δ10-11 (18%)
WT (82%)
Δ10-11 (24%)
WT (76%)
c.917-9T>A
Exon 11 skipping
7-nt insertion
Δ10-11 (16%)
Δ11(34%)
7-nt ins (50%)
Δ10-11 (21%)
Δ11(37%)
7-nt ins (35%)
c.917-3C>G
Δ10-11 (23%)
Δ11 (77%)
Δ10-11 (30%)
Δ11 (64%)
c.1030C>T
4-nt deletion
Δ10-11 (6%)
Δ11 (8%)
4-nt del (81%)
WT (5%)
Δ10-11 (14%)
Δ11 (11%)
4-nt del (71%)
WT (5%)
c.1032+3A>T
Δ10-11 (13%)
Δ11 (87%)
Δ10-11 (18%)
Δ11 (82%)

Exon 12

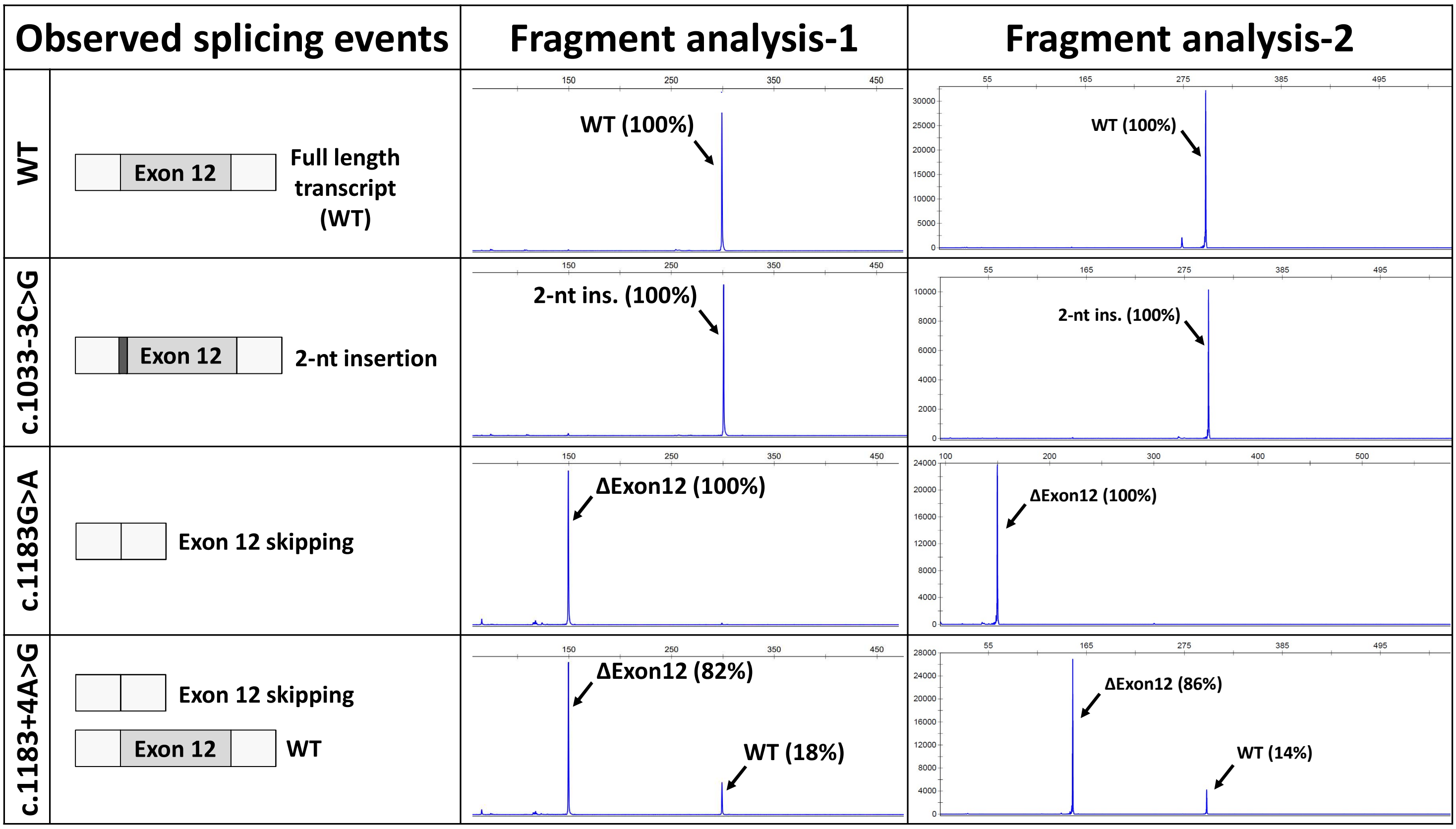

Observed splicing events
Fragment analysis-1
Fragment analysis-2
WT
Exon 12
Full length transcript (WT)
WT (100%)
c.1033-3C>G
2-nt insertion
2-nt ins. (100%)
c.1183G>A
Exon 12 skipping
ΔExon12 (100%)
c.1183+4A>G
ΔExon12 (82%)
WT (18%)
ΔExon12 (86%)
WT (14%)
