# Supplementary material for: Assessing Splicing Variants in the PAX6 Gene: A Comprehensive Minigene Approach
Source: J Cell Mol Med. 2025 Mar 25;29(6):e70459. doi: 10.1111/jcmm.70459 (PMC11936725; doi:10.1111/jcmm.70459)
Supplement: Supplementary file 4 — TABLE S1. Primers used for cloning, mutagenesis and RT‐PCR analysis. [file JCMM-29-e70459-s002.pdf]

Primers for cloning

Minigene	Primer name	Primer sequence 5'->3'
Exon 4	XhoI-PAX6 Ex4F	AAAACTCGAGTTGGGGCATCGCTTCTACTG
	BamHl-PAX6 Ex4R	AAAAGGATCCAGAGGGGGTGTGAGTTACAG
Exon 5 (V2.1)	XhoI-PAX6 Ex5F	AAAACTCGAGGTGTTTATGAAGAGGGGCAGT
	BamHl-PAX6 Ex5R	AAAAGGATCCACTGTTCCCACAATGAATGTCT
Exon 5 (V2.2)	XhoI-PAX6 Ex5-2F	AAAACTCGAGTGGCAGGGGGATGAGGATG
	BamHl-PAX6 Ex5-2R	AAAAGGATCCGAGAGGGCGTTGAGAGTGG
Exon 5 (V2.3)	XhoI-PAX6 Ex5-Bp52F	AAAACTCGAGCTTCTTCCTCTTCACTCTGC
Exon 5 (V2.4)	XhoI-PAX6 Ex5-Bp89F	AAAACTCGAGTTTCTGTTCTTGTTCTCCCTCA
	BamHl-PAX6 Ex5-minR	AAAAGGATCCGGAGGATCACCTGCAGAATT
Exon 6	BglII-PAX6-Ex5F	TTAAGATCTTTGTAGAGCTAGCTCACAGC
	SalI-PAX6-Ex7R	TTAGTCGACCATGCCGTCTGCGCCCATCT
Exon 7	XhoI-PAX6 Ex7F	AAAACTCGAGTTAACAAAGCAAAGCAACCGTG
	BamHl-PAX6 Ex7R	AAAAGGATCCCGCAGCTCGTTCTTATGCAC
Exon 8	PstI-PAX6-int7F	TTACTGCAGTTTTTAAAAGAAGGCTGACAGT
	BamHI-PAX6-int8R	TTAGGATCCTGAAAAGATGCCCAGAGAAAT
Exons 9-11	XhoI-PAX6 Ex9F	AAAACTCGAGAATGCCACATCTTCAGTACAAAG
	XhoI-PAX6 Ex9-2F	AAAACTCGAGATTTCAGGCCACAGAAAGGTC
	BamHl-PAX6 Ex11R	AAAAGGATCCAGGGCATGAATTAATGAGTCAAT
Exon 12	SacI-PAX6 Ex12F	AAAAGAGCTCATCCAGATTTGCAGGGAGAGG
	BamHl-PAX6 Ex12R	AAAAGGATCCTCAACAAGCACGCACCTACAG
Exon 13	XhoI-PAX6 Ex13F	AAAACTCGAGATTGGTTTTCCAGAGACAGCTT
	Xbal-PAX6 Ex13R	AAAATCTAGACTAGTGCATGTTGTTCCAGGT

Primers for mutagenesis

Variant	Primer sequence 5'->3'
c.52G>A	TCGGTGGTGTCTTTGTCAACAGGCGGCCACTGCCGGACTC
c.94C>G	GGCCCCGCTGTGAGCTACCTCTACAATCTTCTGCCGGGTG
c.140A>C	GCCGGGAGGATCACCGGCAGAATTCGGGAAATGTCGC
c.141G>A	GCCGGGAGGATCACttGCAGAATTCGGGAAATGTCGC
c.141+3G>C	GCGGCGCCGGGAGGATGACCTGCAGAATTCGGGAAATGTCG
c.141+4A>T	GCGGCGCCGGGAGGAacACCTGCAGAATTCGGGAAATGTCG
c.142-5T>G	CTGTCCACTTCCCCTAGGCAGGTGTCCAACGGATG
c.142-3C>G	TGTCCACTTCCCCTATGGAGGTGTCCAACGGATGTGTGAG
c.155G>A	CTGCCCAGAATTTTACTCACATATCCGTTGGACACCTGCATAG
c.164A>C	TCCAACGGATGTGTGAGTACAATTCTGGGCAGGTATTACGAGAC
c.233T>C	CGGTGGTAGTAAACCGAGAGCAGCGACTCCAGAAGTTGTAAGC
c.233T>G	CGGTGGTAGTAAACCGAGAGGAGCGACTCCAGAAGTTGTAAGC
c.255C>T	CCGCTTATACTGGGCTATTTTACTTACAACTTCTGGAGTCGCTAC
c.333C>A	GATTACTGTCCGAGGGGGTATGTACCAACGATAACATACCAAGC
c.357+4A>T	CCAACGATAACATACCAAGCGTATGTTCATTGAGAACATCTGCCCTC
c.357+5G>C	CCAACGATAACATACCAAGCGTAACTTCATTGAGAACATCTGCCCTC

c.357+5G>A	CCAACGATAACATACCAAGCGTAAATTCATTGAGAACATCTGCCCTC
c.357+136G>A	CACATTTGTCTCCTTTGTACCTAGGGGAACAGAGAGGAATG
c.357+334G>A	CCAGAACTTTCCTTCAGGTATCACACATCCATTTCCATCCT
c.485G>A	GCACCCGCCCTGGTTAGTATCCGGGGACTTCGGTG
c.681A>G	CTTTGAAAAACTCTATCACCCTTCTCCAGGGCCTCAATTTGC
c.682G>A	CTACTTTGAAAAACTCTATCACTTTTCTCCAGGGCCTCAATTTG
c.683-3C>G	CAATATGGAAAATCAACTTACTCTTTGAGAGTTTGAGAGAACCCA
c.683-5T>C	CAATATGGAAAATCAACTTACTCTCTCAGAGTTTGAGAGAACCCA
c.683-9C>G	CAATATGGAAAATCAACTTAGTCTTTCAGAGTTTGAGAGAACCCA
c.763C>T	TCTACCTGAAGCAAGAATATAGGTACCGAGAGACTGTG
c.764A>G	TCTACCTGAAGCAAGAATACGGGTACCGAGAGACTGTG
c.765G>C	TCTACCTGAAGCAAGAATACACGTACCGAGAGACTGTG
c.765G>T	TCTACCTGAAGCAAGAATACATGTACCGAGAGACTGTG
c.766-3C>G	CTGTCCCACCTGATTTCGAGGTATGGTTTTCTAATCGAAGG
c.770G>A	GCCCTTCGATTAGAAAACTATACCTGGAAATCAGGTGGGA
c.917-3C>G	CTGTCATTTCTCTTGCCCTGAGTTTCCTCCTTCACATCTG
c.917-9T>A	ACTACTGTCATTTCTCTAGCCCTCAGTTTCCTCCTTC
c.1030C>T	ACCAGCCGCACTTACTTACATAGGCAGGTTATTTG
c.1032+3A>T	ACCACCAGCCGCACTAACTTGCATAGGCAGGTTATTTG
c.1033-3C>G	GTTTGCCTCTCTCCTCAGAGCCCCCAGTCCCCA
c.1183G>A	TCGGGCACCACTTCAACAAGTGAGCCACTGCTTTCTGCAGGC
c.1183+4A>G	GCACCACTTCAACAGGTGGGCCACTGCTTTCTGCAGGC

Primers for PCR

Primer name	Primer sequence 5'->3'
TurboFP-F	ACAAAGAGACCTACGTCGAGCA
GFP-R	AGCTCGATCAGCACGGGCACGAT
TurboFP-F_FAM	/6-FAM/-ACAAAGAGACCTACGTCGAGCA
